# Assessing Hardy-Weinberg equilibrium in T2T-aligned 1000 genomes project

**DOI:** 10.1371/journal.pgen.1012280

**Published:** 2026-09-15

**Authors:** Elika Garg, Jaffa Romain, Lei Sun, Andrew D. Paterson

**Affiliations:** 1 Genetics and Genome Biology Program, The Hospital for Sick Children, Toronto, Ontario, Canada; 2 Division of Biostatistics and 4 Division of Epidemiology, Dalla Lana School of Public Health, University of Toronto, Toronto, Ontario, Canada; 3 Department of Statistical Sciences, University of Toronto, Toronto, Ontario, Canada; 4 Epidemiology, Dalla Lana School of Public Health, University of Toronto, Toronto, Ontario, Canada; Huazhong University of Science and Technology Tongji Medical College, CHINA

## Abstract

Quality control of markers in genome-wide association studies often includes testing for Hardy-Weinberg equilibrium (HWE). However, this is usually implemented in a homogeneous population without stratifying by sex. Previous work indicates sex-based selection at numerous autosomal loci in cohorts with active recruitment. Sex chromosome sequences can also interfere with autosomal SNPs. These motivate a re-examination of HWE in sex-aware analyses. Using the telomere-to-telomere (T2Tv2)-aligned high-coverage whole genome sequencing data from 2,490 individuals in the 1000 Genomes Project, we examined genome-wide sex-specific deviations from HWE across five super-populations. Our analyses were restricted to bi-allelic SNPs with non-missing genotypes and minor allele frequency (MAF) ≥5% in both sexes of the five super-populations. We applied an allele-based framework to quantify both the magnitude and direction of Hardy–Weinberg disequilibrium (HWD), followed by a second-order omnibus meta-analysis that combined HWD results across populations and sexes. At a genome-wide significance threshold of p < 5e-8, 0.9% of autosomal SNPs exhibited significant deviations from HWE. The majority of these deviations were associated with genomic features indicative of poor sequence quality. Restricting the analysis to reliable genomic regions substantially reduced the number of signals, yielding 255 autosomal SNPs and one non-pseudoautosomal chromosome X SNP. Among these, 140 autosomal SNPs displayed significant heterogeneity across populations but not across sexes. Notably, eight SNPs within a 15-bp region on chromosome 14q31.3 showed excess heterozygosity in both sexes of the African super-population (AFR). Finally, we developed a multivariate predictor of HWD based on sequence features, providing a practical tool that can be integrated into existing quality control pipelines for whole genome sequencing studies.

## Author summary

We conducted a specific quality control test, which compares the observed and expected genotype counts, on an updated version of the 1000 Genomes Project whole genome sequence data generated on ~2500 individuals. We first performed this analysis by grouping the data by ancestry and sex. We then combined and contrasted the group results. We found that most regions that differed between observed and expected counts overlapped regions of the genome that are difficult to sequence using current short-read technology. In the remaining regions, we found an interesting cluster of SNPs in a single ancestry group, where there is a large excess of heterozygous genotypes. GWASes typically use a standard strict threshold for this quality control test for genotyping arrays to remove SNPs. Here, we suggest a more nuanced approach that is applicable to whole genome sequence data.

## Introduction

### Hardy-Weinberg equilibrium (HWE)

HWE is a fundamental principle in population genetics that quantifies the relationship between allele and genotype frequencies [1,2]. A SNP is in HWE if the genotype frequencies depend only on the allele frequencies in a given population. Deviation from HWE can arise from technical factors or violations of HWE assumptions, such as mutation, migration, non-random mating, or selection [3]. HWE is particularly important in the quality control of GWAS, where deviation from HWE can raise concerns about data quality, selection, or underlying population structure. However, not all deviation is artefactual: it can be driven by disease association [4,5], participation bias [6] or admixture [7]. Graffelman et al., 2017 performed a genome-wide study of HWE in the Japanese and Yoruban populations from the phase 3 1000 Genomes Project (1KGP), which was aligned to GRCh37, and they reported more HWE deviation than would be expected by chance alone. They also indicated there is a complex relationship between read depth and deviation from HWE, where most of the SNPs were derived from low-coverage WGS. Apart from that, there has been surprisingly little emphasis on HWE in 1KGP, despite the use of this data for many purposes which often rely upon the assumption of HWE [8,9].

### Sex-specific alignment of 1000 Genomes Project (1KGP) data using the complete T2T reference genome

Recently, there was a release of 1KGP consisting of 3,202 high-coverage short-read whole genome sequenced samples aligned to GRCh38 (GCA_000001405.15; [9]). This data has been re-aligned to the Telomere-to-Telomere reference genome; first to T2T-CHM13v1.0 [10,11], excluding chromosome Y (chrY), and then to T2T-CHM13v2.0, including chrY [12]. We refer to the latter re-aligned version as “v2”. However, neither of these re-aligned versions of 1KGP have been examined extensively for HWE. Given the purported advantages of v2, we chose to use this realignment for our work. Here we describe HWE in v2 with methods previously used by our group with a particular focus on sex differences.

### Genotyping errors and reliable regions

Data aligned to T2T has been shown to improve autosomal mappability and variant calling compared to GRCh38 [11]. However, aligning short-read sequencing data to a reference genome in a sex-chromosome agnostic manner can lead to misalignments. This is because sex chromosomes have a common evolutionary origin and consequently share regions of high sequence similarity [12], for example, the pseudo-autosomal regions (PARs). To reduce misalignments, in v2, XYalign [13] masks chromosome Y of the reference genome for XX samples, and chromosome Y PARs for XY samples [12].

In general, however, systematic sequencing and alignment biases can lead to genotyping errors and interfere with true biological signals in genetic data [14]. Common errors in short-read sequencing arise when reads align to multiple genomic regions with high sequence similarity, leading to incorrect SNP calls [15]. For example, autosomal sequences can misalign to chromosome Y in males, leading to sex differences in genotyping errors due to a paralogous sequence variant (PSV; [16,17]). Such misalignment can also occur due to segmental duplications, low-complexity regions and repeats. The Genome in a Bottle consortium (GIAB) has categorized certain genomic regions as “difficult-to-sequence” regions, where confidence in calls is low [18]. In contrast, Illumina has defined short-read accessibility masks to mark regions where SNP discovery and genotyping are relatively reliable [19]. Ogata et al., 2023 have comprehensively assembled other lists of problematic sequence features which, if excluded, can improve the accuracy of biological signals [20]. We have combined several of these genomic features to create a set of “reliable regions”, which we use as a filter prior to our main analyses.

### Sex differences in minor allele frequency (sdMAF)

Recent studies have reported sdMAF of SNPs on, but not limited to, chromosome X (chrX; [6,17,21]). sdMAF on chrX in PARs and at PAR-NPR boundaries are likely due to sex-linkage [17,22]. But sdMAF can also be driven by genotyping errors, for example, PSVs causing cross-alignment of genomic regions between autosomes and sex chromosomes [17].

Pirastu et al., 2021 performed an autosomal GWAS for sex using data from 2.46 M customers of 23andMe, having previously shown that sex was an autosomally heritable trait in studies with active, as opposed to passive, participation. They identified 158 autosomal loci associated with sex and examined them further. The follow-up analysis included HWE testing (p < 10e-6), but it was performed only in sex-combined samples, despite the observed sdMAF. Combining groups with different MAFs can lead to Hardy–Weinberg disequilibrium (HWD). This motivates the current work to analyze sex-stratified HWD.

### HWE in recently-admixed populations

In recently-admixed populations, theoretical analyses have demonstrated that achieving HWE on chrX requires more than one generation, unlike the autosomes [7,23,24]. However, this has never been demonstrated in practice. Since 1KGP includes some recently-admixed populations (e.g., African Ancestry in the Southwest US [ASW] and African Caribbean in Barbados [ACB]), we compare HWD between autosomes and NPR in these populations as an additional analysis.

**Fig 1:**
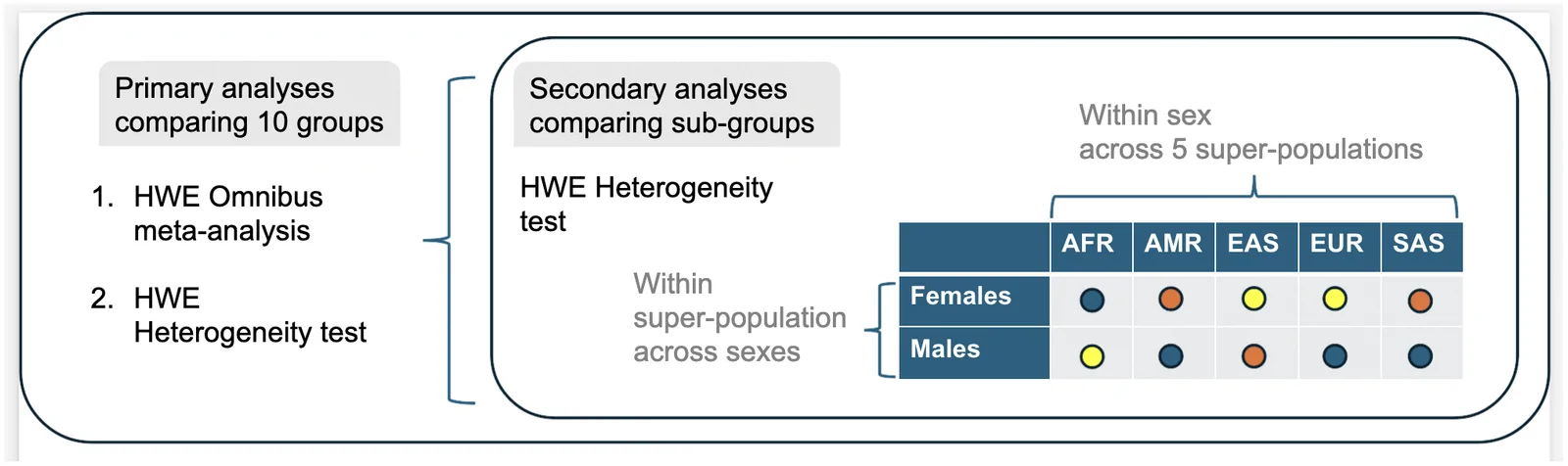
Representative diagram of analyses. A diagram shows the structure of primary and secondary analyses. Primary analyses compare 10 groups and include both HWE omnibus meta-analysis and HWE heterogeneity test. Secondary analyses test for HWE heterogeneity within sub-groups, for example, 5 super-populations within a sex or sexes within a super-population.

In conclusion, although HWE evaluation has been extensively used for quality control in large-scale genotyping/sequencing studies, it has not been previously studied in high-coverage short-read whole genome sequences from 1KGP aligned to T2T-CHM13v2.0. Here, we perform sex- and super-population-stratified HWE analyses in this data, and we then combine the HWD results using meta-analysis, including analysis of heterogeneity (Fig 1). SNPs identified as deviating from HWE were then subjected to bioinformatic annotations. Additionally, we used these annotations to generate a multivariate predictor of HWD.

## Results

### 1000 Genomes project data

The high coverage 1KGP data which has been re-aligned in a sex-specific manner to the T2T-CHM13v2.0, referred to here as “v2”, consists of 2,490 unrelated samples (1,263 females and 1,227 males) of which a total of 11 million bi-allelic non-missing SNPs were first selected irrespective of MAF (10,984,307 in autosomes, 383,435 in NPR, 16,217 in PAR1, and 1,006 in PAR2). Table 1 shows the number of males and females in each of the five super-populations (ranging from 170 males in AMR to 336 females in AFR), along with the number of SNPs with MAF ≥ 5% in each group (ranging from 4,987,954 in EAS males to 7,918,101 in AFR females).

**Table 1:** Number of samples and SNPs with MAF ≥ 5% in each of the 10 groups. Groups are made on the basis of super-population (SAS = South Asian, EAS = East Asian, EUR = European, AMR = Admixed American, AFR = African) and sex (F = females, M = males). Note that SNP counts in females include NPR, which is absent in males.

Super-population	Sex	No. of samples	No. of SNPs with MAF ≥ 5%
SAS	F	227	5,720,041
	M	257	5,552,906
EAS	F	260	5,156,954
	M	244	4,987,954
EUR	F	263	5,569,928
	M	240	5,453,535
AMR	F	177	5,735,361
	M	170	5,588,358
AFR	F	336	7,918,101
	M	316	7,648,566

### HWD stratified by sex and super-population and meta-analyses

Genome-wide significant deviations from HWE as measured by p < 5e-8, were found across the genome in all five sex-stratified super-populations.

#### HWD in autosomes.

**Fig 2:**
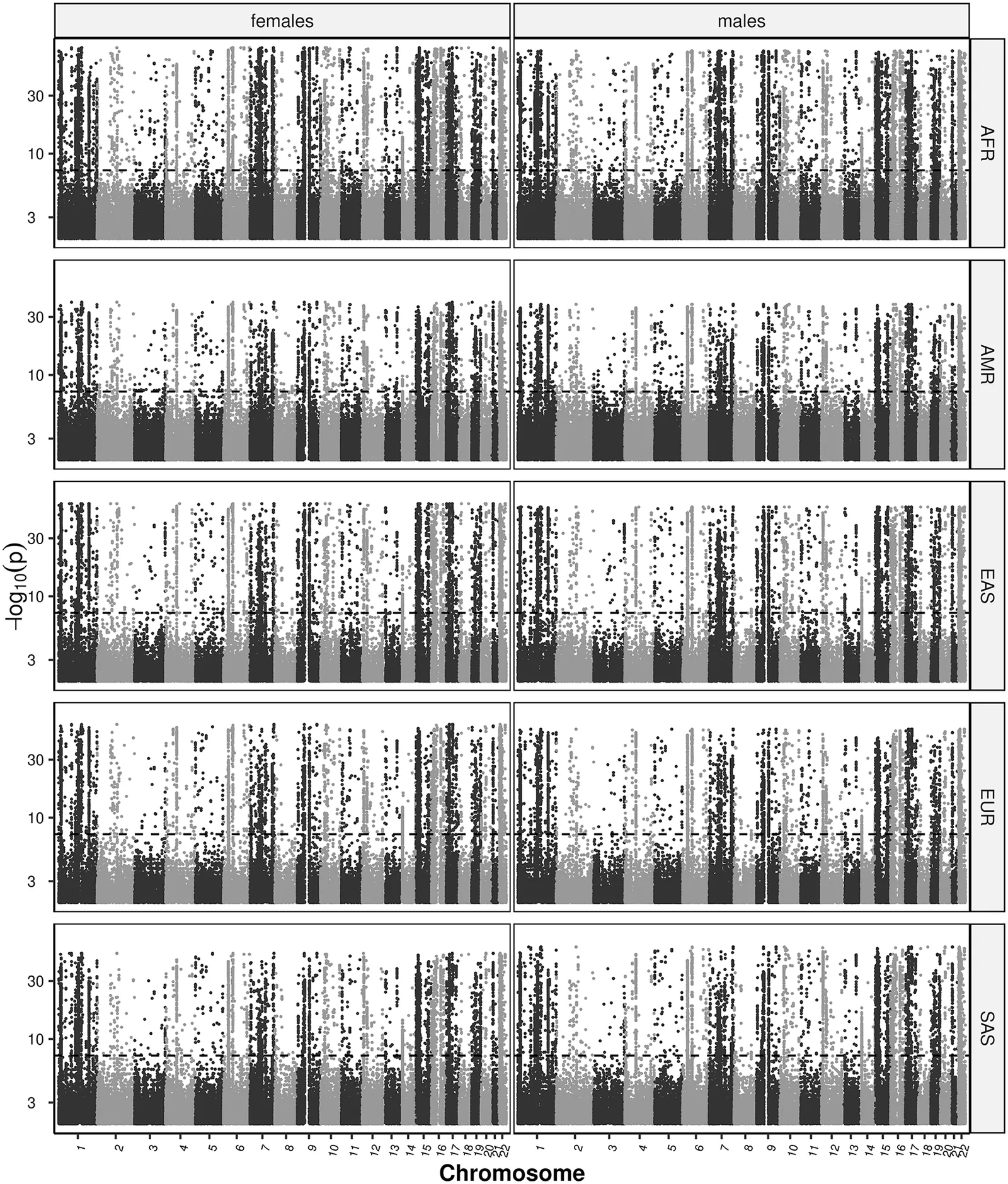
Manhattan plots for autosome-wide HWE in each of the 10 groups. HWE test results from five super-populations and two sexes are shown for autosomal SNPs with MAF ≥ 5%. The y-axis is presented on -log10 scale for SNPs with p-value<0.01. The dashed line represents genome-wide significance threshold of p = 5e-8.

Fig 2 shows the HWE test results in each of the 10 groups (5 super-populations x two sexes) in autosomal SNPs with MAF ≥ 5% in each group (see Table 1). HWD in the autosomes had similar patterns across groups, for example, at telomeres and around centromeres.

**Fig 3:**
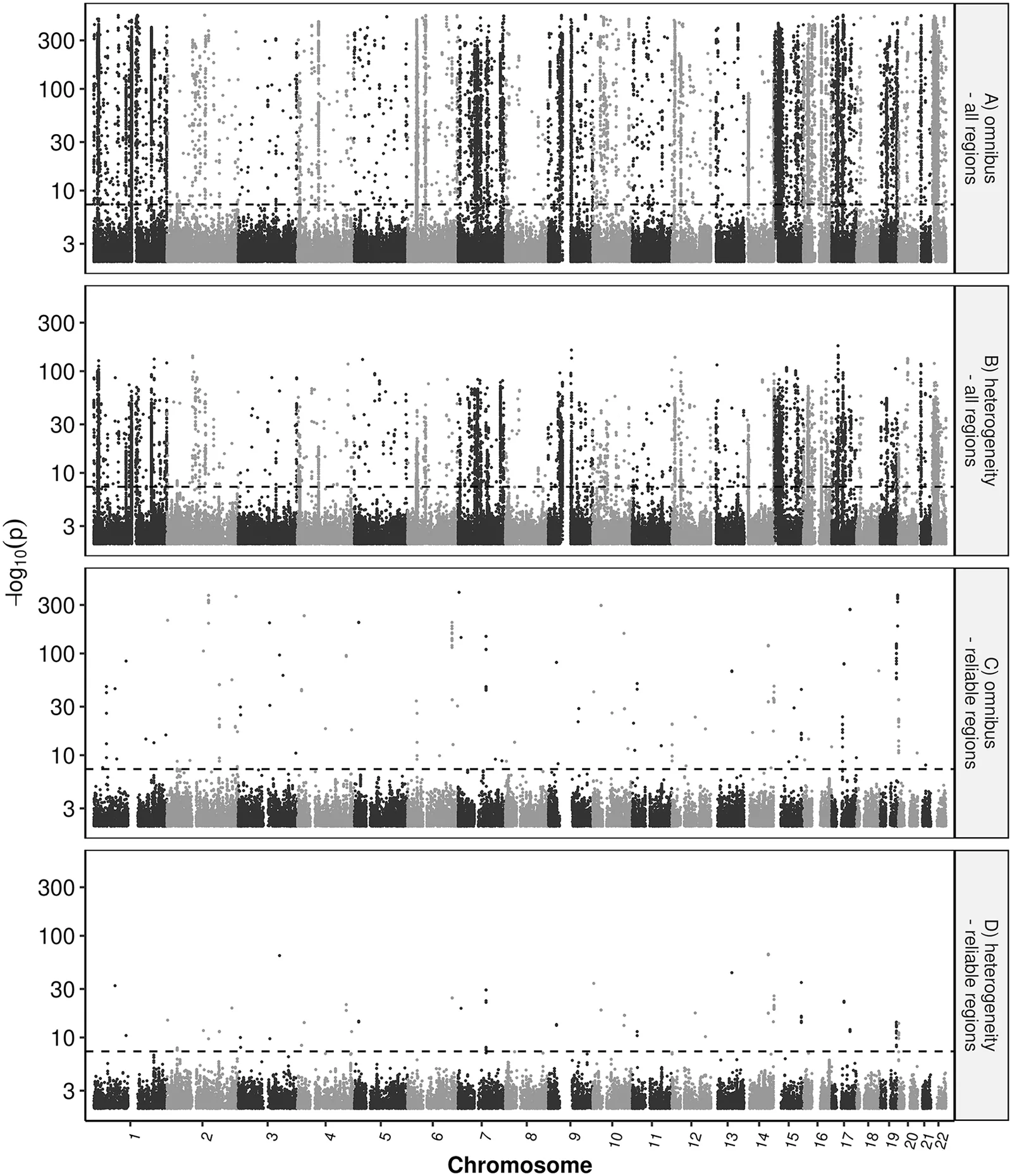
Manhattan plots for autosome-wide HWE omnibus meta-analysis and HWE heterogeneity of 10 groups. Test results from five super-populations and two sexes are shown for autosomal SNPs which have MAF ≥ 5% in all groups: A) HWE omnibus meta-analysis and B) HWE heterogeneity results for all regions (n = 3,377,201), C) HWE omnibus meta-analysis and D) HWE heterogeneity results for reliable regions (n = 1,347,995). The y-axis is presented on -log10 scale for SNPs with p-value<0.01. The dashed line represents genome-wide significance threshold of p = 5e-8.

Fig 3A shows the HWE test results from the omnibus meta-analysis of all 10 groups for 3,377,201 autosomal SNPs (0.9% SNPs are significant at p < 5e-8; Table 2) that are in common at MAF ≥ 5% in each group. Fig 3B shows the HWE heterogeneity results for the same set of SNPs. Table 2 presents the overlap between SNPs identified in the HWE omnibus and heterogeneity meta-analyses, stratified by the significance of each test (p < 5e-8). About half of the significant SNPs in the omnibus meta-analysis also showed significant HWE heterogeneity, which signifies unequal contribution of groups (discussed in more detail below in: “SNPs with omnibus HWD in reliable regions”). Interestingly, seven SNPs exhibited significant HWE heterogeneity but were not significant in the omnibus test, as expected when directional effects differ across groups. These SNPs mapped to two loci on chromosomes 2 and 4 (Tables 2 and S1). At the region on chr2, the most pronounced deviation from HWE was observed in AMR females, characterized by a paucity of heterozygotes, and the HWE heterogeneity was driven by super-populations within females. In contrast, in the region on chr4, the most pronounced deviation from HWE was observed in AFR males, characterized by an excess of heterozygosity (S1 Table).

**Table 2:** Overlap of SNPs between HWE omnibus meta-analysis and HWE heterogeneity comparing 10 groups. Test results from five super-populations and two sexes are compared for autosomal SNPs with MAF ≥ 5% in all groups. Numbers in brackets indicate the subset of SNPs in reliable regions.

Tests in autosomal SNPs on 10 groups		HWE Omnibus Meta-Analysis (reliable)		Total
		significant	not-significant	
**HWE Heterogeneity (reliable)**	significant	14,982 (140)	7 (3)	14,989 (143)
	not-significant	15,956 (115)	3,346,256 (1,347,737)	3,362,212 (1,347,852)
**Total**		30,938 (255)	3,346,263 (1,347,740)	3,377,201 (1,347,995)

#### HWD in chromosome X.

**Fig 4:**
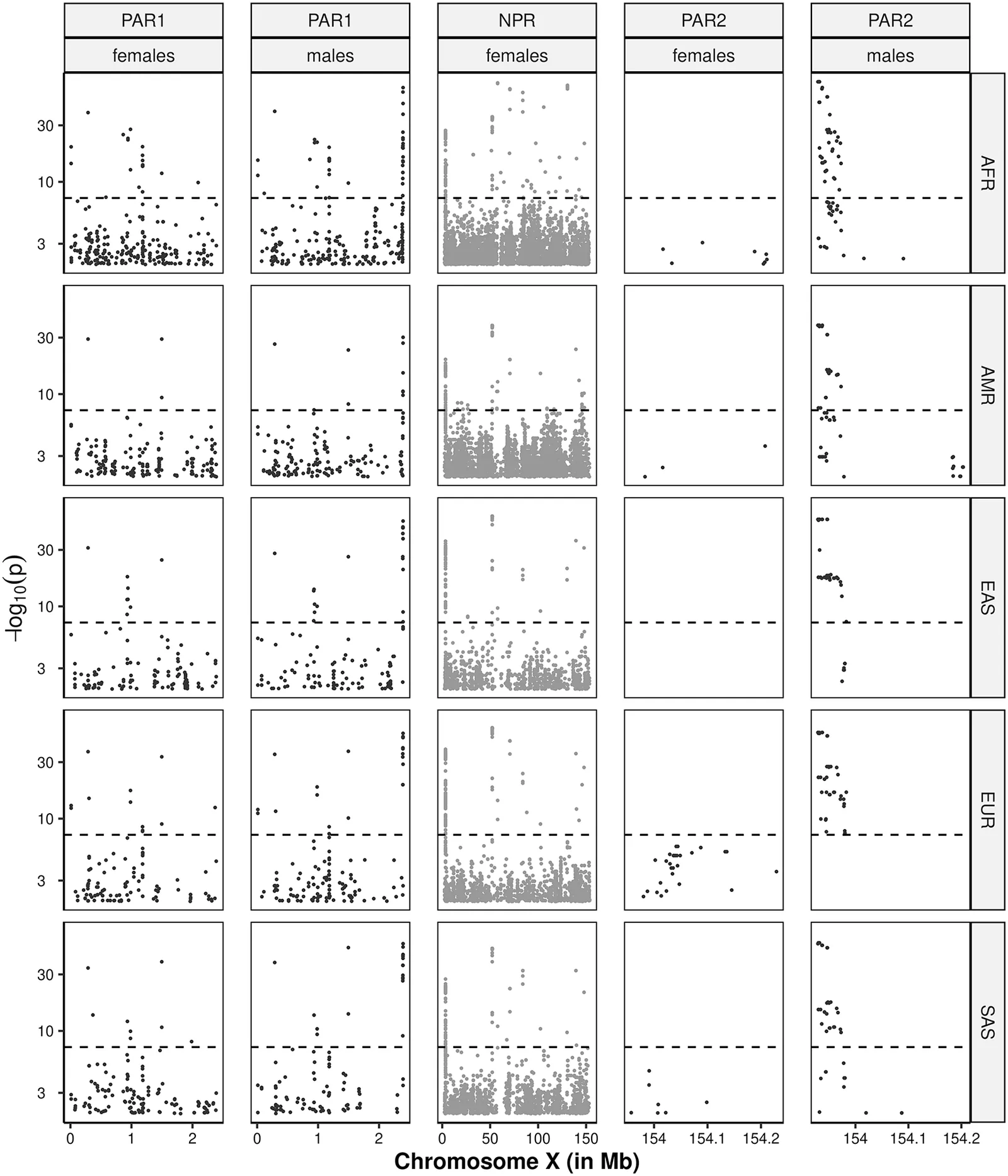
Manhattan plots for X chromosome HWE in each of the 10 groups. HWE results from five super-populations and two sexes are shown for chrX SNPs with MAF ≥ 5%. NPR data is only for females. The y-axis is presented on -log10 scale for SNPs with p-value<0.01. The dashed line represents genome-wide significance threshold of p = 5e-8.

Similarly, Fig 4 shows the HWE test results for chromosome X in each of the 10 groups. There is high HWD across super-populations: (i) at the centromeric boundaries of both PAR1 and PAR2 in males, and (ii) in the NPR region close to PAR1 in females.

**Fig 5:**
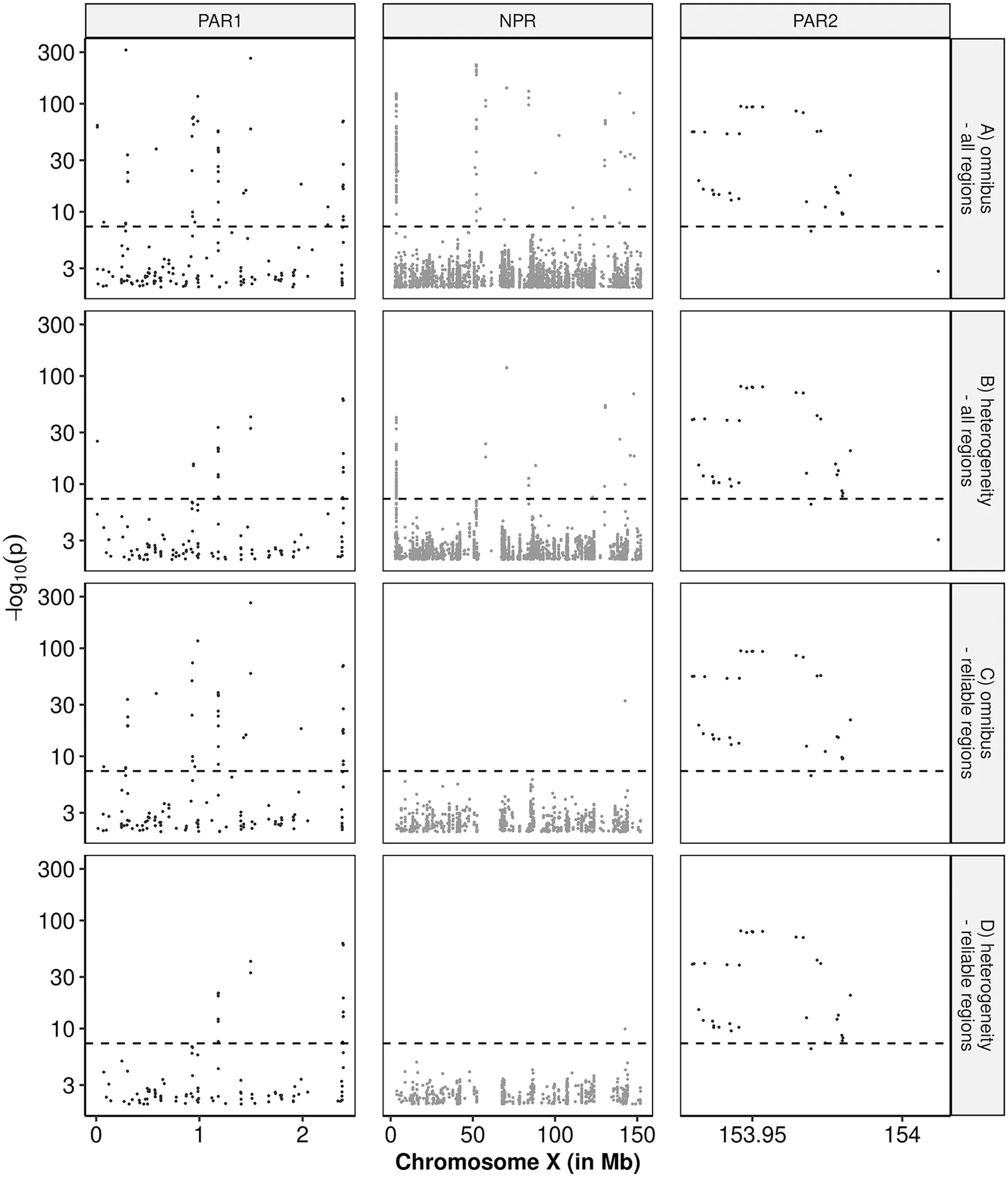
Manhattan plots for X chromosome HWE omnibus meta-analysis and HWE heterogeneity of 10 groups. Test results from five super-populations and two sexes are shown for chrX SNPs which have MAF ≥ 5% in all groups: A) HWE omnibus meta-analysis and B) HWE heterogeneity results for all regions (PAR1 = 5,381, PAR2 = 277, NPR = 97,225), C) HWE omnibus meta-analysis and D) HWE heterogeneity results for reliable regions (PAR1 = 4,473, PAR2 = 252, NPR = 26,890). NPR data is only for females. Genomic features were not available for PARs except difficult-to-sequence regions. The y-axis is presented on -log10 scale for SNPs with p-value<0.01. The dashed line represents genome-wide significance threshold of p = 5e-8.

Fig 5A shows the HWE test results from the omnibus meta-analysis of SNPs with MAF ≥ 5% in all groups for chromosome X. Across all 10 groups, there were 5,381 PAR1 and 277 PAR2 SNPs, as well as 97,225 NPR SNPs in the 5 female groups. Fig 5B shows the corresponding HWE heterogeneity results.

In the omnibus meta-analysis, 1% SNPs on the autosomes were in HWD, compared to 1.5% and 0.3% SNPs on the PARs and NPR, respectively. In the HWE heterogeneity meta-analysis, 0.44% SNPs on the autosomes were in HWD, compared to 0.97% and 0.07% SNPs on the PARs and NPR, respectively. Accompanying Figs 2-5, S1-S4 Figs show the corresponding QQ plots and histograms of HWE testing p-values.

#### Sex differences in HWD (sdHWD).

Additionally, a heterogeneity test measuring sdHWD within each super-population yielded 80 unique genome-wide significant SNPs (24 in autosomes, 20 in PAR1 and 36 in PAR2); all 80 SNPs had MAF ≥ 5% in both sexes of a super-population.

### Effect of genomic features on HWD

Certain regions of the genome are prone to misalignment in short-read sequencing, leading to genotyping errors. We evaluated the effects of these genomic features on HWD patterns and defined “reliable regions” as those with positive features and without negative features (see Methods).

**Fig 6:**
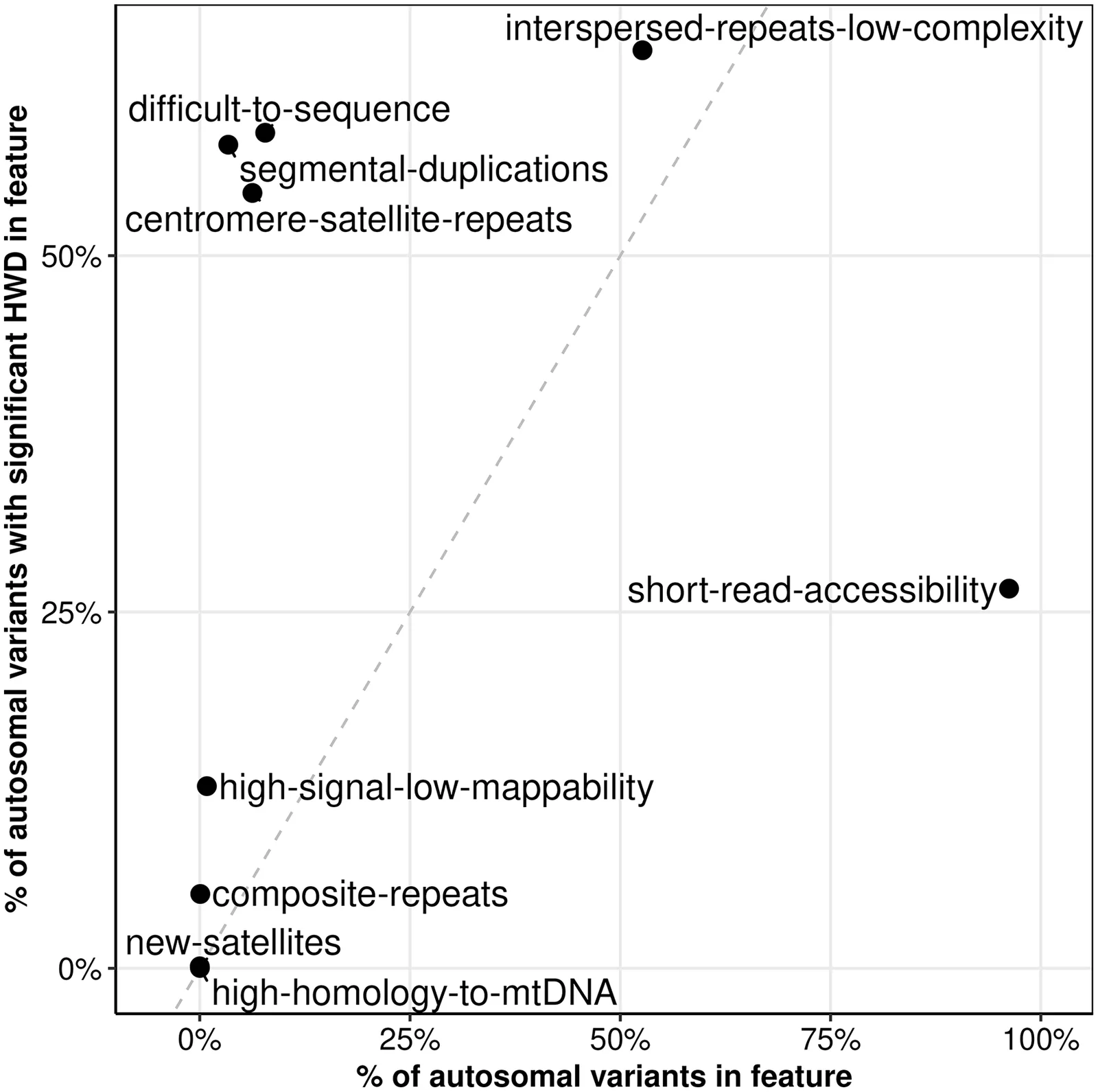
Relationship between HWD and genomic features for autosomal SNPs. The scatter plot compares the percentage of SNPs in a feature (out of 3,377,201 SNPs) to the percentage of SNPs in that feature with significant HWD (out of 30,938 SNPs). Two genomic features (“assembly-gaps” and “telomere”) had no overlaps with the analyzed genome and hence are not displayed here. The dashed line represents the identity line.

Fig 6 shows the enrichment of negative genomic features in autosomal SNPs with HWD. Over half of the SNPs in three negative features (difficult-to-sequence regions, segmental duplications and centromere/satellite regions) had significant HWD, even though they constituted less than 10% of the autosomal SNPs. In contrast, fewer than a third of SNPs in the positive feature (short-read accessibility mask) showed significant HWD, despite covering 96% of the autosomes. When the negative features were combined, they accounted for 96% of SNPs with significant HWD. S2 Table describes the number of SNPs and the χ^2^_1_ test for each of these features.

#### HWD in autosomes.

Fig 3C shows the distribution of HWE p-values from the omnibus meta-analysis after restricting positions to reliable regions in the autosomes (1,347,995 SNPs, which is 40% of unrestricted autosomal SNPs analyzed above). Restricting positions reduced the autosomal SNPs with genome-wide significant HWD from 1% to 0.02% (from 30,938–255 SNPs). Fig 3D shows the corresponding HWE heterogeneity results.

There were 56 significant SNPs with no adjacent SNPs within a 100kb-interval that were also genome-wide significant; however, a few clusters of significant SNPs emerged after the omnibus meta-analysis.

#### HWD in chromosome X.

In parallel, Fig 5C shows the distribution of HWE p-values from the omnibus meta-analysis after restricting positions to reliable regions on chromosome X (26,890 in NPR, 4,473 in PAR1 and 252 in PAR2). Restricting PAR positions increased the proportion of SNPs in HWD from 1.54% to 1.59% because the only available genomic feature for PARs was difficult-to-sequence regions (the other features were not available for the PARs). However, restricting NPR positions to reliable regions reduced the SNPs in HWD from 0.3% to a single SNP (rs859902). Fig 5D shows the corresponding HWE heterogeneity results.

### SNPs with omnibus HWD in reliable regions

The 255 autosomal SNPs with HWD in reliable regions (as shown in Fig 3C) fell into 90 distinct 100 kb non-overlapping intervals. S3 Table provides genomic details for the 256 SNPs (255 on autosomes and 1 on NPR) after lifting over to GRCh38 for functional annotations. Five autosomal SNPs were not present on GRCh38, and an additional 15 had failed QC in the original work, which was aligned to GRCh38 [9]. S4 Table provides the NHGRI-EBI GWAS catalog results for the 10 SNPs with GWAS p < 5e-8 (see S5 Table for a summary of the number of studies associated with each SNP). Three out of these 10 SNPs were in the major histocompatibility complex (MHC) region on 6p21 and were associated with multiple blood cell traits. All 10 SNPs were found in studies primarily based on European populations, likely reflecting the gross over-representation of European populations in the GWAS catalog [25].

#### Evidence for HWE heterogeneity.

S6 Table provides group-wise allele counts and HWD results for the 256 SNPs, and S7 Table reports their meta-analysis results. Of the 255 autosomal SNPs with significant omnibus HWD, 140 exhibited significant heterogeneity in HWD across the 10 groups (Table 2). S7 Table also includes results from heterogeneity tests conducted within sexes across super-populations, and within super-populations between sexes. Overall, as expected, there was more consistency between sexes of the same super-population than between super-populations of the same sex. Of the 140 SNPs, 79 and 86 exhibited significant heterogeneity in HWD across the 5 super-population groups in females and males, respectively (including 58 in common). However, none of the 140 SNPs were significant across sexes in super-population groups. Additionally, 34 of the 140 SNPs were only significant in the heterogeneity test of HWD when all 10 groups were analysed together.

**Fig 7:**
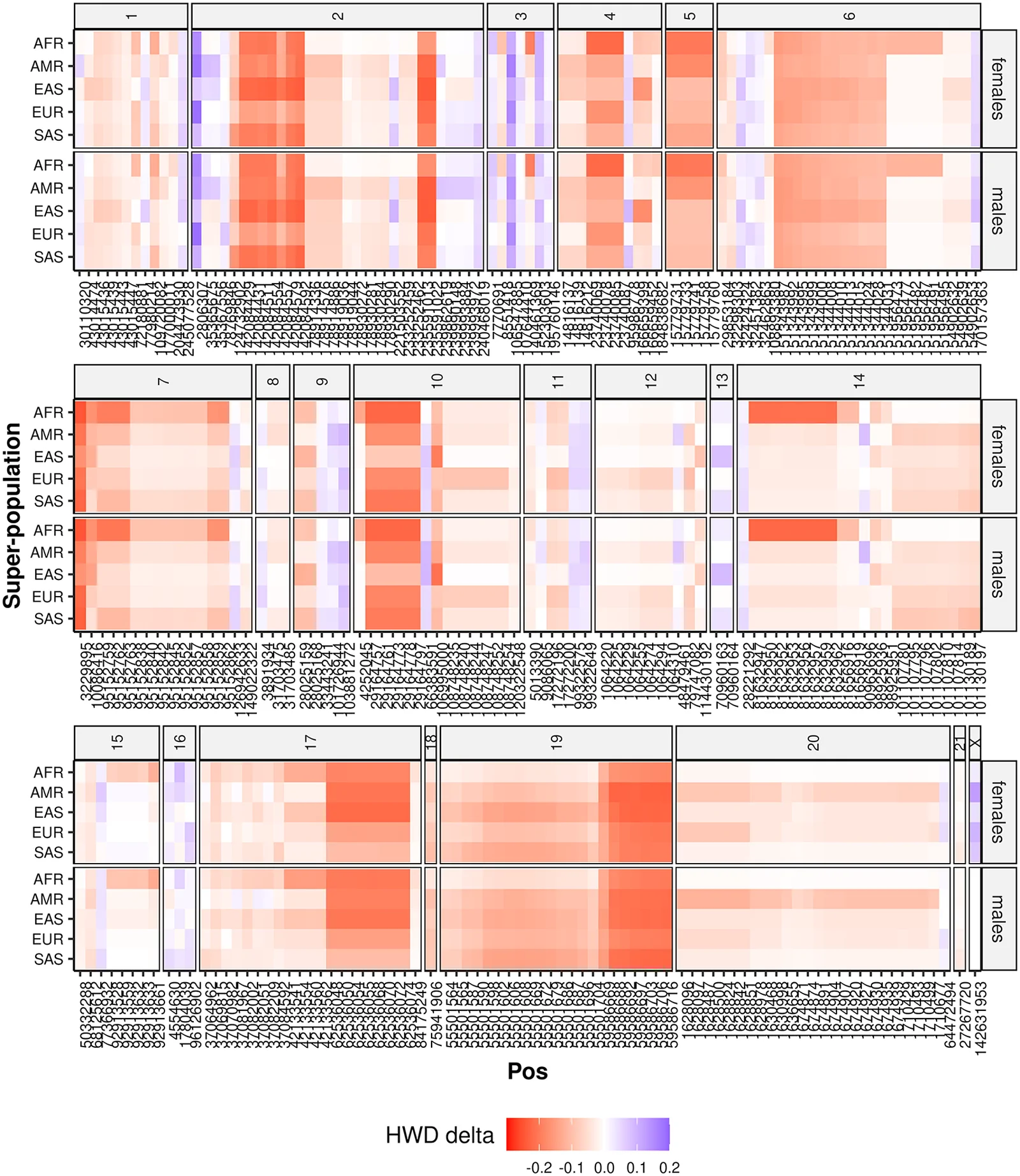
Heatmap of HWD delta for 256 SNPs with significant HWD. HWD delta is shown by a signed color scale for each of the 10 groups at the 256 SNPs (S7 Table). Panels represent chromosomes and genomic location, with each chromosome arranged in ascending order on the X-axis. Red gradient represents a negative delta, which means an excess of heterozygotes. See companion S5 Fig for non-gradient colors.

Fig 7 shows the HWD delta across groups, generally consistent, with exceptions, such as at the chr14 region described below. Of the 256 SNPs, 200 have a negative HWD delta in all groups (S5 Fig), indicating an excess of heterozygotes. 53 of 256 SNPs had significant HWD within all groups, and another 35 SNPs had significant HWD only in the AFR male and female groups (S6 Fig).

#### Interesting sequence on chromosome 14q31.3.

**Fig 8:**
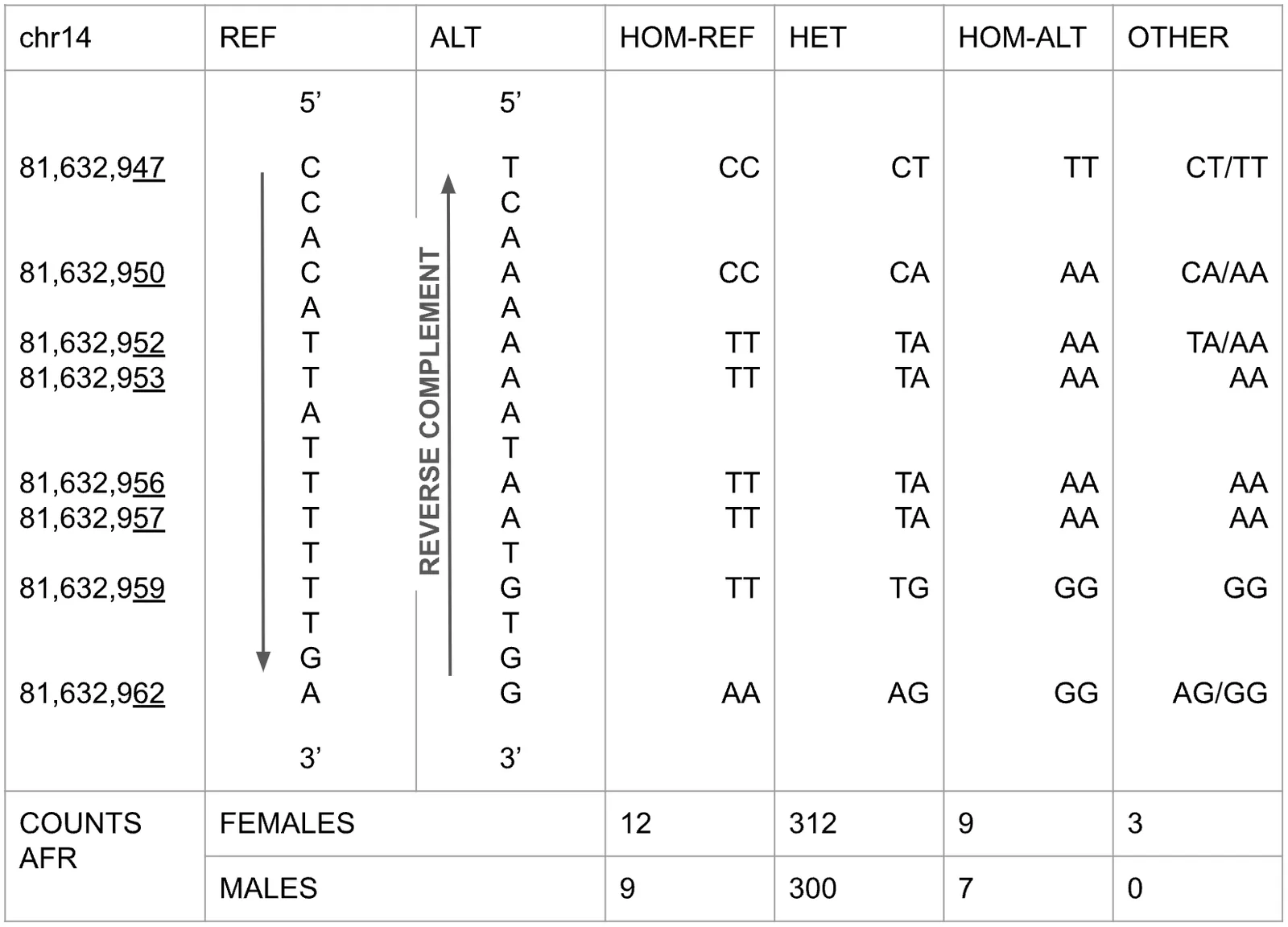
AFR genotype counts at the chr14 locus. The alternate sequence over a 15 bp region at chr14:81,632,947-81,632,962 is the reverse complement of its reference sequence (see S7 Fig). This region consists of 8 SNPs where HWD was observed (see S7 Table). These 8 SNPs have high LD and excess heterozygotes in both male and female AFR samples, as shown by the haplotypes formed by their diploid genotype counts.

Notably, an interesting sequence of eight SNPs was observed over a 15-bp region (chr14:81,632,947–81,632,962 at 14q31.3), where the reference and alternate sequences were reverse complements of each other (S7 Fig). HWD at this region was driven by heterozygosity excess in both sexes of the AFR super-population, where over 90% of individuals were heterozygous at all 8 SNPs (Fig 8). In contrast, non-AFR populations showed no significant HWD at this region (S7 Table). These SNPs passed QC in the original work, which was aligned to GRCh38 [9]. Allele bias at this region for the AFR super-population is high both in v2 (S8 Fig) as well as in GRCh38 (S9 Fig), with estimates of 5:1 allele depth across sexes and SNPs. This could indicate unrecognized repetitive variation at this region in AFR samples, such as a VNTR, making genotyping challenging. Additional quality parameters in v2 are provided in S10 Fig.

Two other regions are of note (S7 Table and Fig 7): (i) 7 SNPs at chr20:1,628,096-1,628,851 in intron 1 of *SIRPB1* with high HWD in both AMR males and females, and EUR females; (ii) 7 SNPs at chr19:59,586,669–59,586,716 with excess heterozygosity in all 10 groups (HWD delta ranging from -0.12 to -0.23).

#### HWD in PARs.

S8 Table provides meta-analysis results for 44 SNPs in PAR1 and 31 SNPs in PAR2, which have significant omnibus HWD after removing difficult-to-sequence regions. In contrast to the autosomes, the PARs exhibited greater similarity in HWD patterns across super-populations than between sexes, essentially because males showed higher HWD near the PAR–NPR boundaries. At the boundary of PAR1 and NPR (2-2.5Mb on chromosome X), 9 SNPs were omnibus HWD significant (Fig 5C), of which 7 also demonstrated significant heterogeneity in HWD. Further analysis indicated that all of them were heterogenous across super-populations within males. Moreover, 5 of them were heterogenous between sexes in AFR, 2 in EAS and 1 in EUR, with all showing significant deviation only in males (Fig 4). Similarly, in PAR2, 31 SNPs were omnibus HWD significant (Fig 5C), and all of them also showed significant heterogeneity, with HWD present in males.

#### sdHWD.

No autosomal sdHWD SNPs were observed in reliable regions. After removing difficult-to-sequence regions, no PAR1 SNPs and only one PAR2 SNP remained; this SNP (chrX:153,977,745) had sdHWD in the EUR super-population and showed a significant sdMAF (S9 Table).

### Multivariate genomic feature model for autosomal HWD identified by the omnibus test prior to restricting to the reliable regions

Many features associated with HWD (S2 Table) showed correlation (S11-12 Figs). As expected, “short read accessibility”, a positive feature, was negatively correlated with others. Additionally, features with fewer SNPs, such as “new satellites” or “high homology to mtDNA”, showed little correlation with others.

Five features were selected based on their importance (S13 Fig) and AIC to be used in a multivariate model. To account for linkage disequilibrium among nearby SNPs, we retained the first SNP in each 100 kb non-overlapping interval for both the training set (odd autosomes: 13,524 SNPs of which 190 had significant HWD) and the test set (even autosomes: 13,358 SNPs of which 103 had significant HWD). The model predicted HWD significance status with high accuracy (AUC is 99.4%; S10 Table and S14 Fig). Although this model was trained and tested on different chromosomes of the same data, different chromosomes are generally accepted to be independent.

### Comparison of autosomal and NPR HWD in recently-admixed populations

Because recent admixture can result in HWD, we examined this. In recently-admixed populations (ASW and ACB) from the AFR super-population, we examined HWE separately for autosomes (79 females and 71 males) and the chrX NPR (79 females) stratified by MAF (SNPs in reliable regions at each MAF bin: 339,046 in 0.1 ≤ MAF < 0.2, 330,291 in 0.2 ≤ MAF < 0.3, 315,586 in 0.3 ≤ MAF < 0.4 and 308,859 in MAF ≥ 0.4). Inspection of QQ plots (S15 Fig) revealed that SNPs in NPR did not deviate from HWE, whereas SNPs in most MAF bins in the autosomes had inflated HWD (except 0.1 ≤ MAF < 0.2). All 78 significant SNPs (p < 5e-8) are in the autosomes, and overlap with the 255 SNPs in the primary HWE omnibus meta-analysis (S7 Table). Inspection of their HWD delta statistics (S16 Fig) reveals that most SNPs exhibit heterozygote excess (S11 Table), following a similar pattern to the 255 SNPs (Fig 8).

## Methods

### Data

We used high-coverage whole genome sequences from the 1000 Genomes Project aligned to T2T-CHM13v2.0 using XYalign [12], which we refer to as “v2”. To our knowledge, this data has not been subjected to quality control based on HWE thresholds. SNPs were filtered after applying VQSR (v4.1.9.0) using known sites lifted over from GRCh38 (as described at https://github.com/schatzlab/t2t-chm13-chry). We performed further quality control on the data by removing up to second-degree relatives and selecting bi-allelic SNPs with non-missing genotypes. We analyzed both autosomes (chromosomes 1–22) and sex chromosomes (chromosomes X and Y). The two pseudo-autosomal regions (PAR1 and PAR2) of sex chromosomes were studied in all individuals. The non-pseudo-autosomal region (NPR) of chromosome X was only studied in females, as the concept of HWE does not apply to males in NPR.

For the primary HWD analyses, we used 2,490 unrelated samples (1,263 females and 1,227 males) from v2. We divided these individuals by super-population and sex to form 10 groups (5 super-populations x 2 sexes) for all analyses (except the recent-admixture analysis), which was conducted on SNPs with MAF ≥ 5% in each of the 10 groups (Fig 1).

For the secondary analysis of HWD in recently-admixed populations, we used 150 unrelated samples (79 females and 71 males) from v2 that belonged to the ASW (African Ancestry in Southwest US) and ACB (African Caribbean in Barbados) populations. Here, due to a smaller sample size, we analyzed SNPs with MAF ≥ 10% in the 150 samples.

### HWE

#### Quantifying HWD.

We quantify departure from Hardy–Weinberg equilibrium (HWE), i.e., Hardy-Weinberg disequilibrium (HWD), as 𝛿, the frequency difference between one of the observed homozygous genotypes and its expectation under HWE in a given population/group [26]. Specifically,


$$\begin{array}{c} \delta =p_{AA}-p_{A}^{\text{2}}=p_{\text{2}}-p^{\text{2}}, \end{array}$$

where $p_{AA}$ is the AA genotype frequency and $p_{A}$ the A allele frequency. 𝛿 = 0 indicates HWE and 𝛿 < 0 indicates excess heterozygosity.

#### Test statistics for one group.

The test statistic takes the general form,


$$\begin{array}{c} Z=\hat{\beta }/\hat{\sigma }, \end{array}$$

where 𝛽 and 𝜎 are context-dependent parameters, and $\hat{\beta }$ and $\hat{\sigma }$ are their estimates.

When testing for HWD,


$$\begin{array}{c} \hat{\beta }=\hat{\delta }={\hat{p}}_{\text{2}}-{\hat{p}}^{\text{2}}, \end{array}$$

$$\begin{array}{c} {\hat{\sigma }}^{\text{2}}=\frac{{\hat{p}}^{\text{2}}{\left(\text{1}-\hat{p}\right)}^{\text{2}}}{n}, \end{array}$$

and the resulting test statistic $T_{D}$ is,


$$\begin{array}{c} T_{D}=\frac{{\hat{\delta }}^{\text{2}}}{{\hat{\sigma }}^{\text{2}}}=Z^{\text{2}}. \end{array}$$

Under the null hypothesis of HWE, $T_{D}$ is asymptotically chi-squared distributed with one degree of freedom, $\chi_{\text{1}}^{\text{2}}$. Although $T_{D}$ does not have the usual Pearson chi-square form, it is equivalent to the traditional test [27]. Males were excluded from NPR analyses because HWE does not apply to NPR variants in males.

#### Meta-analyzing HWD across groups.

For group k, k=1,…K , let


$$\begin{array}{c} Z_{k}=\hat{\beta_{k}}/\hat{\sigma_{k}}. \end{array}$$

To test for HWD across the K  groups, the classical meta-analysis is suboptimal because it uses the weighted average of $Z_{k}$ and is therefore sensitive to the direction of 𝛽’s (i.e., of 𝛿’s). A priori one group may show excess heterozygosity (𝛿 < 0) while another shows depletion (𝛿 > 0). We therefore use an omnibus meta-analysis aggregating $Z_{k}^{\text{2}}$ [28,29]. The resulting statistic $T_{M}$ appears below and is asymptotically $\chi_{K}^{\text{2}}$ distributed under the null of HWE in all populations.


$$\begin{array}{c} T_{M}=\sum_{k=\text{1}}^{K}Z_{k}^{\text{2}}. \end{array}$$

#### Heterogeneity tests.

To assess between-group differences in effect size (β), we use Cochran’s Q ,


$$\begin{array}{c} T_{Q}=\sum_{k=\text{1}}^{K}Z_{k}^{\text{2}}-\frac{\text{1}}{\sum_{k=\text{1}}^{K}{\left(\frac{\text{1}}{\hat{\sigma_{k}}}\right)}^{\text{2}}}\cdot {\left(\sum_{k=\text{1}}^{K}\frac{\text{1}}{\hat{\sigma_{k}}}Z_{k}\right)}^{\text{2}}, \end{array}$$

which is asymptotically $\chi_{K-\text{1}}^{\text{2}}$ distributed under the null of no heterogeneity. We apply $T_{Q}$ to evaluate HWD heterogeneity across the 10 groups (five super-populations and two sexes). For selected SNPs, we also evaluate HWD heterogeneity across super-populations within sexes, or between sexes within super-populations (Fig 1).

For each super-population, we first test for sex differences in HWD (sdHWD). We then compare MAFs between the sexes (sdMAF; [17]), noting that the magnitude of HWD depends on MAF: $-p^{\text{2}}\leq \delta \leq p(\text{1}-p)$ [27]. We note that when K=2  as in sdHWD and sdMAF analyses, $T_{Q}$ reduces the familiar two-sample comparison with group-specific variance estimates,


$$\begin{array}{c} T_{Q}={\left(\frac{\hat{\beta_{\text{1}}}-\hat{\beta_{\text{2}}}}{\sqrt{\hat{{\sigma_{\text{1}}}^{\text{2}}}+\hat{{\sigma_{\text{2}}}^{\text{2}}}}}\right)}^{\text{2}}. \end{array}$$

For sdMAF on autosomal and PAR SNPs we use,


$$\begin{array}{c} {\hat{\beta }}_{k}={\hat{p}}_{sex}, \end{array}$$

$$\begin{array}{c} {\hat{\sigma }}_{k}^{\text{2}}=\frac{{\hat{p}}_{sex}\left(\text{1}-{\hat{p}}_{sex}\right)+{\hat{\delta }}_{sex}}{\text{2}n_{sex}}. \end{array}$$

The sdMAF statistic for NPR SNPs differs slightly [17]; we omit it here because we only analyze SNPs for which HWD testing is possible in males.

### Genomic features

Short-read next-generation sequencing has difficulty accurately calling variants in certain genomic regions, which can affect HWE results. Therefore, we created a list of “reliable regions” using predefined annotations. We used several published annotations available for the T2T-CHM13 reference genome provided by a) the “Telomere-to-Telomere” (T2T) Consortium: i) centromere/satellite repeats [30], ii) composite repeats [31], iii) segmental duplications [32], iv) new satellites [30], v) interspersed repeats and low complexity regions [31]; b) Ogata et al., 2023: vi) high signal and low mappability regions, vii) regions of high homology to mtDNA (nuclear mitochondrial DNA), viii) assembly gaps, ix) telomeres; c) Genome-In-A-Bottle: x) difficult to sequence regions [18]; d) Illumina: xi) short-read accessibility mask [19]. See S12 Table for more details.

All features except for the short-read accessibility mask are “negative” features. Thus, we created a set of “reliable regions” by removing them from the “positive” short-read-accessibility mask. Two genomic features (“assembly gaps” and “telomeres”) were a subset of other features and did not have unique SNPs. No genomic features were available for PARs, except “difficult to sequence regions”.

### Variant annotations

We used the Entrez Programming Utilities (edirect v22.0) to query dbSNP (build 156) to find rsIDs, variant class, and clinVar significance of a SNP.

We used the Ensembl Variant Effect Predictor REST API (v15.8; https://rest.ensembl.org/vep/human/id/{rsid}) to find the most severe consequence of a SNP.

We queried the GWAS catalog APIs with different parameters and merged all results in order to be comprehensive: i) https://www.ebi.ac.uk/gwas/rest/api/singleNucleotidePolymorphisms/{rsid}/associations?projection=associationBySnp, ii) http://www.ebi.ac.uk/gwas/summary-statistics/api/associations/{rsid}?p_upper=5e-8, iii) http://www.ebi.ac.uk/gwas/summary-statistics/api/associations/{rsid}?size=100. The GWAS catalog was queried in June 2024, at which time it was mapped to GRCh38.p14 and dbSNP build 156.

### Predictive model

We created a logistic regression model using five binary genomic features to predict autosomal HWD significance status. We selected the features using the MASS::stepAIC (v7.3-59) function in R (v4.3.0). SNPs in linkage disequilibrium (LD) can unduly affect the model and are often pruned in an ancestry-specific manner. However, given our multi-ancestry data, we opted for a simpler approach: we selected the first SNP in each 100 kb window on every autosome, since LD typically decays at that physical distance [8]. We assigned all odd autosomes to the training set and all even autosomes to the test set.

## Discussion

This study examined Hardy-Weinberg equilibrium in high-coverage whole genome sequencing data from 1KGP aligned to the T2T-CHM13v2.0 reference, with a particular focus on sex- and population-stratified analyses. Across the genome, we found that most apparent deviations from HWE were strongly associated with genomic features linked to poor sequence quality. Restricting analyses to reliable genomic regions substantially reduced the number of significant signals, suggesting sequence-aware filtering can be applied to whole genome sequencing data. Of the remaining SNPs with significant omnibus HWD, about half exhibited significant heterogeneity across super-populations. We performed an in-depth analysis of one of these regions on chr14, where HWD is driven by the AFR super-population.

### Motivations

A key motivation for this work was the recognition that standard HWE testing often relies on statistical significance alone, without considering the magnitude or direction of deviation [33] or the presence of multiple heterogeneous groups [34]. In datasets stratified by ancestry and sex, such simplifications can obscure important structure. Some methods, such as RUTH (Robust Unified Test for Hardy–Weinberg Equilibrium) [35] and others [28], account for heterogeneous groups. It is well known that ignoring ancestry or population strata can inflate false-positive rates in HWE testing. RUTH addresses this problem by modelling ancestry-adjusted HWE deviation using individual-specific allele frequencies and a global deviation parameter, while also incorporating genotype uncertainty through genotype likelihoods. In contrast, our approach follows the omnibus stratified framework of Troendle and Yu (1994), aggregating squared stratum-specific HWE statistics, $Z_{k}^{\text{2}}$, rather than the signed $Z_{k}$. This avoids cancellation when different groups deviate from HWE in opposite directions, for example, when one group shows excess heterozygosity ($\delta_{k}<\text{0}$) and another shows heterozygote depletion ($\delta_{k}>\text{0}$). Although RUTH included a stratified meta-analysis comparator, that comparator used a sample-size-weighted Stouffer Z-score approach and did not evaluate the Troendle and Yu omnibus approach. Thus, unlike RUTH, our approach estimates signed group-specific HWD measures, $\;\delta_{k}$’s, and uses them both for second-order omnibus and heterogeneity testing across groups. This provides interpretable group-specific effect sizes, allowing us to distinguish shared HWD from population- or sex-specific HWD.

We chose the 1KGP because it is a relatively large, publicly available and widely used resource. The vast majority of analyses of 1KGP, including previous HWE analyses [3], utilized the Phase 3 data, which relies primarily on low-coverage whole genome sequencing aligned to GRCh37. Instead, we used high-coverage whole genome sequence data aligned to T2T-CHM13v2.0, which is larger (additional ~200 Mb) and has previously been shown to improve variant calling [11]. Of note, the analysis presented here identified 5 SNPs with HWD that were present in T2T but these SNPs were not present in GRCh38 (S3 Table). Even though current GWASes may not be based on newer reference builds, future arrays, imputations or sequencing will likely be.

We performed sex- and super-population-stratified HWD analysis on this data. We included sex stratification in our analysis because recent work [6,17,21,36] has shown sex differences in allele frequency at a small proportion of SNPs. When we examined sex differences in HWD, we identified only 80 SNPs with significant sdHWD; the majority were located in PARs, and only one survived the restriction to reliable regions. Understanding the diverse contributors to HWD across populations is particularly important, as neglecting these influences can lead to inappropriate application of quality control strategies [7,36,37].

Since the magnitude of HWD delta (δ) depends on MAF, we applied a strict MAF threshold before analyzing our data, which may not be necessary in larger studies. For example, recent studies examining heterozygosity excess [38] or homozygosity deficit [39] have focused predominantly on autosomal SNPs with low MAF in GRCh38-aligned data from multiple studies and technologies. Abramovs et al., 2020 restricted their analysis to protein-coding SNPs in gnomAD 2.1.1 with MAF < 0.05. Oddsson et al., 2023 exclusively analyzed European populations from multiple cohorts and included both imputed and sequenced data to specifically study protein-altering SNPs. Another important limitation of our work is that we examined only bi-allelic SNPs with no missing data that did not overlap with indels. Although multi-allelic extensions of HWE have been developed (e.g. [40]) for microsatellites, we did not implement them here for simplicity.

### Effect of genomic features on HWD

In our work, we emphasize the effect of unreliable regions on HWD. We found that, on the autosomes, restricting positions to reliable regions reduced the proportion of SNPs in HWD from 1% to 0.02%. In fact, three negative genomic features (difficult-to-sequence regions, segmental duplications, and centromere/satellite regions) that encompassed less than 10% of all autosomal SNPs accounted for a large proportion of significant HWD results. This is also supported by multivariate model results, in which these three features are the most significant predictors of HWD. No genomic features were available for PARs, except “difficult to sequence regions”.

HWD patterns in all groups are similar, with deviation typically occurring at telomeres and around centromeres in autosomes (Fig 2; note that chromosomes 13–15, 21, 22 are acrocentric).

### Interesting sequence on chromosome 14q31.3

One notable region with population-specific deviation was at chr14q31.3, where HWD was observed in the AFR superpopulation across 8 SNPs spanning a 15-bp region. Interestingly, the reference and alternate haplotypes are reverse complements of each other (S7 Fig). This raises the possibility that genotypes from this region are not being called properly, which may be due to several factors. Sequence misalignment can result in erroneous genotypes. Others have shown that unappreciated segmental duplications can cause gross heterozygote excess [11]. Technical artifacts like sample contamination can also lead to systematic genotyping errors [41,42]; Byrska-Bishop et al., 2022 used a threshold of 2% to eliminate contaminated samples. Long-read sequencing technology can also cause technical artifacts such as foldbacks (duplicated inversions; [43]).

However, the high linkage disequilibrium among these 8 SNPs is not limited to the data we analyzed; it has also been observed in the pangenome, which includes long-read and linked-read sequencing data for some samples from 1KGP (see Resources and S17 Fig; [44]). Because there were only 24 individuals of African ancestry in the pangenome dataset, we did not perform a statistical analysis of HWD. Moreover, the reference sequence is identical in T2T CHM13v2.0 and GRCh38, even though T2T CHM13v2.0 is of European origin [10], whereas GRCh38 is predominantly of African-European origin [45,46]. Also, the ratio of allele depths for these 8 SNPs follows the same pattern in data aligned to both T2T CHM13v2.0 and GRCh38 (S9 Fig). This region has no alternate contigs in GRCh38.

We note that this region exhibits a high density of variation in dbSNP (build 157, S18 Fig) and overlaps with two multi-nucleotide variants (MNVs, rs796238959 and rs770667319, on GRCh38.14; S19 Fig). rs796238959 (chr14:87412183–87412205; ss1808029058) is derived from the Western African Pygmy population [47]. rs770667319 (chr14:87412188–87412200; ss1576679280) is derived from the GenomeDenmark project [48]. MNVs can give rise to alignment challenges [49], and impact variant calling. These SNPs are intronic in all four reported transcripts of LINC02296 (long intergenic non-protein coding RNA gene). We also note that the unusual structure of reference and alternate haplotypes in this region resembles that of an inversion heterozygote [50]. Moreover, given that the allele depth ratio in the AFR super-population is 4.8, it is possible that this region contains structural variation, but it has not been reported (S12 Table). Future analysis of additional pangenome samples may provide insight into deviation in such regions.

### HWE in recently-admixed populations

Separately, we examined the impact of recent admixture on HWD on chrX. Theoretically, it takes more generations for HWE to be achieved on chrX compared to the autosomes [23,24], but this has not been tested. Therefore, we used two recently-admixed populations (ASW and ACB) in our analysis. We compared HWD in NPR and autosomes stratified by MAF. Contrary to the theoretical expectation, our results indicate that whereas 78 of 1,347,995 autosomal SNPs deviate from HWE, none of the 26,890 NPR SNPs do. As expected, we observed an excess of heterozygotes in the SNPs that deviate from HWE (S16 Fig) [7]. This is in contrast to what we expect when two sub-populations have non-random mating which results in paucity of heterozygosity (i.e., Wahlund effect) [51]. Relatively recent admixture and/or non-random mating can result in heterozygote excess on autosomes. In addition, African American populations have been shown to have higher African ancestry on chrX owing to the sex bias in ancestry contributions brought about by asymmetric mating [52]. Our analysis has a limited scope due to small sample sizes, especially on chrX, and deserves attention in future larger populations including other admixed groups.

### Limitations

A general limitation of this work is that the sample size is modest, and SNPs were restricted (biallelic SNPs with MAF ≥ 5% and no missingness). The statistic ${TDT}_{DTD}$ relies on an asymptotic $\chi_{\text{1}}^{\text{2}}$ approximation, which may be inaccurate when MAF is low, or the sample size is small. Exact HWE tests avoid this asymptotic approximation and can provide better finite-sample calibration, although exact p-values are not necessarily more conservative than $\chi^{\text{2}}$ p-values for every observed dataset. In this study, we reduced this concern by restricting the primary analyses to SNPs with MAF ≥ 5% in all 10 groups (super-populations x sexes), but finite-sample calibration may still be imperfect for variants near the MAF threshold. We retained $T_{D}$ because it is based on the signed HWD measure δ, which allows us to distinguish excess heterozygosity from heterozygote depletion and to perform second-order omnibus and heterogeneity analyses across groups. Exact tests may therefore be useful as complementary sensitivity analyses, particularly for rare variants or smaller strata.

HWD patterns for other variant types, which were not examined here, may differ. Short-read whole genome sequencing from cell-lines can have more artifacts [9], which may be resolved by using long-read sequencing of non-transformed cells. Although we trained and tested the multivariate model on different chromosomes, the underlying data was the same. Currently, due to a lack of other large short-read sequencing data aligned to v2, we have not tested it on an external dataset.

### Recommendations

Our analysis reveals that males have high HWD at PAR boundaries, indicating that applying sex-specific HWE-based quality control in males in PARs may inadvertently remove SNPs. We found SNPs with marked deviations, despite filtering out regions that are not ‘reliable’ using several external genomic annotations. Therefore, this filtering may not be sufficient. The annotations may also be biased by the ancestral composition of genomes sequenced to date.

### Resources

WGS data (‘v2’): https://s3-us-west-2.amazonaws.com/human-pangenomics/index.html?prefix=T2T/CHM13/assemblies/variants/1000_Genomes_Project/chm13v2.0/unrelated_samples_2504/

WGS data (GRCh38):

http://ftp.1000genomes.ebi.ac.uk/vol1/ftp/data_collections/1000G_2504_high_coverage/working/20201028_3202_raw_GT_with_annot/

1KGP ancestries:

https://projecteuclid.org/journals/annals-of-applied-statistics/volume-17/issue-2/Leveraging-HardyWeinberg-disequilibrium-for-association-testing-in-case-control-studies/10.1214/22-AOAS1695.short

UCSC genome browser result for chr14 locus on GRCh38 (Multiple alignments of ‘Human Pangenome - HPRC’ track can be turned on): https://genome.ucsc.edu/cgi-bin/hgTracks?db=hg38&lastVirtModeType=default&lastVirtModeExtraState=&virtModeType=default&virtMode=0&nonVirtPosition=&position=chr14%3A87412182%2D87412206&hgsid=2500872119_ZhA4jGydieyrY2C5Mn02OWsx0S0p

UCSC genome browser result for chr14 locus on T2T CHM13v2.0: https://genome.ucsc.edu/cgi-bin/hgTracks?db=hub_3671779_hs1&lastVirtModeType=default&lastVirtModeExtraState=&virtModeType=default&virtMode=0&nonVirtPosition=&position=chr14%3A81632947%2D81632962&hgsid=3326470641_djDKIWfSW1n98DvnDjd76A7gmlSu

## Supporting information

S1 Fig QQ plots and p-value histograms for autosome-wide HWE in each of the 10 groups. HWE results from five super-populations and two sexes are shown for autosomal SNPs with MAF ≥ 5% (GC λ for females: AFR = 1.12, AMR = 1.28, EAS = 1.11, EUR = 1.08, SAS = 1.17; males: AFR = 1.10, AMR = 1.32, EAS = 1.10, EUR = 1.10, SAS = 1.14).
(PNG)

S2 Fig QQ plots and p-value histograms for autosome-wide HWE meta-analysis of 10 groups. HWE omnibus meta-analysis results from five super-populations and two sexes are shown for autosomal SNPs which have MAF ≥ 5% in all groups and fall in: a) all regions (GC λ =  1.38) b) reliable regions (GC λ = 1.33).
(PNG)

S3 Fig QQ plots and p-value histograms for X-chromosome HWE in each of the 10 groups. HWE results from five super-populations and two sexes are shown for chrX SNPs with MAF ≥ 5% (GC λ for females: AFR =  1.19, AMR = 1.56, EAS = 1.11, EUR = 1.12, SAS = 1.27; males: AFR = 1.11, AMR = 1.36, EAS = 1.18, EUR = 1.00, SAS = 1.09). NPR data is only for females.
(PNG)

S4 Fig QQ plots and p-value histograms for X-chromosome HWE meta-analysis of 10 groups. HWE omnibus meta-analysis results from five super-populations and two sexes are shown for chrX SNPs which have MAF ≥ 5% in all groups and fall in: a) all regions (GC λ = 1.55) b) reliable regions (GC λ = 1.44). NPR data is only for females. Genomic features were not available for PARs except difficult-to-sequence regions.
(PNG)

S5 Fig Heatmap of HWD delta for 256 SNPs with significant HWD. HWD delta is shown by a signed color scale for each of the 10 groups at the 256 SNPs (S7 Table). Panels represent chromosomes and genomic location with each chromosome arranged in ascending order on the X-axis. Red color represents negative delta, which means excess of heterozygotes. See companion Fig 7 for gradient colors.
(PNG)

S6 Fig Upset plot for 256 SNPs with significant HWD. HWD significance in each of the 10 groups is compared across the 256 SNPs (S7 Table). Sets are arranged alphabetically and intersections are arranged by frequency.
(PNG)

S7 Fig Reverse complement sequence at chr14 locus. The reference and alternate sequences at the chr14:81,632,947–81,632,962 position are reverse complements of each other. SNPs that are highlighted with the last two digits of the base pair positions are the 8 SNPs that were observed to have HWD in our analysis.
(PNG)

S8 Fig Allele depth at chr14 locus in T2T compared to neighbouring HWD SNPs. Ratio of alternative and reference allele depth is shown for the chr14 locus (chr14:81,632,947–81,632,962) and neighbouring SNPs with HWD (locations in S7 Table) in the 10 groups. Dashed line indicates a ratio of 0.5. Y-axis is presented on -log10 scale.
(PNG)

S9 Fig Allele depth at chr14 locus in T2T compared to GRCh38. Ratio of alternative and reference allele depth is shown for the chr14 locus (chr14:81,632,947–81,632,962) on T2T (top) with corresponding location on GRCh38 (bottom) in the 10 groups. Dashed line indicates a ratio of 0.5. Y-axis is presented on a log10 scale.
(PNG)

S10 Fig Quality parameters of chr14 locus in T2T compared to neighbouring HWD SNPs. Site-specific parameters are shown for the chr14 locus (chr14:81,632,947–81,632,962 in blue) and neighbouring SNPs with HWD (locations in S7 Table).
(PNG)

S11 Fig Correlation between autosome-wide genomic features. Pairwise Spearman correlation between genomic features in the autosome is shown by a signed color scale. Correlation between each feature and significant HWD status is also shown in the bottom row.
(PNG)

S12 Fig Upset plot for autosome-wide genomic features. Overlap of genomic feature positions is examined across analyzed autosomal SNPs (n = 3,377,201). They map on to 3,325,907 unique positions; 51,294 positions are not covered by any of the genomic features examined. Sets are arranged alphabetically and intersections are arranged by frequency; top 20 intersections are shown.
(PNG)

S13 Fig Variable importance of genomic features in the predictive autosome-wide model. Variable importance is shown for: a) 9 features in training set, b) 5 selected features in training set, c) 9 features in test set, d) 5 selected features in test set.
(PNG)

S14 Fig ROC curve for the predictive autosome-wide model of genomic features. A multivariate logistic model to predict binary HWD significance status was trained on odd autosomes and tested on even autosomes.
(PNG)

S15 Fig QQ plots for autosomes and NPR HWE of recently-admixed groups. HWE results from two populations (recently-admixed groups ASW and ACB) are shown for autosomal and chrX SNPs with MAF ≥ 10%. NPR data is only for females. SNPs are stratified by MAF (GC λ for autosomes, chrX): 0.1 ≤ MAF < 0.2 (1.02,1.09), 0.2 ≤ MAF < 0.3 (1.07,1.11), 0.3 ≤ MAF < 0.4 (1.01,1.18), MAF ≥ 0.4 (1.00,0.89).
(PNG)

S16 Fig Histogram of HWD delta of significant SNPs in recently-admixed groups. HWE results from two populations (recently-admixed groups ASW and ACB) are shown for autosomal SNPs with MAF ≥ 10% and HWE p-value < 5e-8 (S11 Table).
(PNG)

S17 Fig UCSC genome browser at chr14 locus. Pangenome variation at the chr14 locus is examined in the UCSC genome browser (link in Resources).
(PNG)

S18 Fig Variation viewer at chr14 locus. A high density of variation is seen at the chr14 locus in dbSNP (build 157) Variation Viewer.
(PNG)

S19 Fig MNVs at chr14 locus. Alignment of the two MNVs (rs796238959 and rs770667319) at chr14 locus.
(PNG)

S1 Table SNPs with significant HWE heterogeneity but not-significant HWE omnibus test results. HWE omnibus meta-analysis, HWE heterogeneity and sdMAF results are shown for 7 SNPs (from Table 2) along with group-specific details, and a column to indicate if they fall in reliable regions.
(XLSX)

S2 Table HWD in autosome-wide genomic features. Impact of each genomic feature on autosomal HWD is shown by chi-squared test and odds-ratio. Two genomic features (“assembly gaps” and “telomere”) had no overlaps with the analyzed genome and hence are not displayed here.
(XLSX)

S3 Table Genomic details of genome-wide SNPs with HWD in reliable regions. 255 SNPs on autosomes and 1 on NPR with significant HWD were found in reliable regions. Their coordinates were lifted over to GRCh38 for functional annotations from dbSNP and Ensembl VEP (see Methods). An additional annotation indicates their QC status from the original work [2].
(XLSX)

S4 Table GWAS catalog results of genome-wide SNPs with HWD in reliable regions. 10 SNPs on autosomes had a significant result in the NHGRI-EBI GWAS catalog with p < 5e-8.
(XLSX)

S5 Table Summary of GWAS catalog results of genome-wide SNPs with HWD in reliable regions. The number of studies associated with each of the 10 SNPs with p < 5e-8 in the NHGRI-EBI GWAS catalog.
(XLSX)

S6 Table Group-wise details of genome-wide SNPs with HWD in reliable regions. Allele counts, MAF and HWE results are shown for the 256 SNPs in each of the 10 groups.
(XLSX)

S7 Table Meta-analysis results of genome-wide SNPs with HWD in reliable regions. HWE omnibus meta-analysis, HWE heterogeneity and sdMAF results are shown for the 256 SNPs along with group-specific details from S6 Table.
(XLSX)

S8 Table Meta-analysis of SNPs with HWD in PARs after removing difficult-to-sequence regions. HWE omnibus meta-analysis, HWE heterogeneity and sdMAF results are shown for 44 PAR1 and 31 PAR2 SNPs along with group-specific details (similar to S7 Table).
(XLSX)

S9 Table Meta-analysis and group-wise details results of one SNP with sdHWD in PAR2. HWE omnibus meta-analysis, HWE heterogeneity and sdMAF results are shown for one PAR2 SNP along with sex-specific details for the EUR super-population group where it was found.
(XLSX)

S10 Table Results for the multivariate genomic feature model for autosomal HWD. A multivariate logistic model to predict binary HWD significance status was trained on odd autosomes and tested on even autosomes.
(XLSX)

S11 Table HWE results of significant SNPs in recently-admixed populations. HWE results from two populations (recently-admixed groups ASW and ACB) are shown for autosomal SNPs with MAF ≥ 10% and HWE p-value < 5e-8 (S16 Fig).
(XLSX)

S12 Table Sources for the genomic features used in analysis. Resource table for genomic features with links to the resources. Last column indicates if that genomic feature was available for PARs.
(XLSX)
