# Supplementary material for: Assessing Hardy-Weinberg equilibrium in T2T-aligned 1000 genomes project
Source: PLoS Genet. 2026 Sep 15;22(9):e1012280. doi: 10.1371/journal.pgen.1012280 (PMC13596830; doi:10.1371/journal.pgen.1012280)
Supplement: Supplementary file 1 [file pgen.1012280.s033.pdf]

Please see our responses in blue below.
We have made the following structural changes:

- Added page breaks to tables to improve readability in the current format.
- Removed figures from text, and supplied as separate files, with legends at the end of the manuscript.
- Added sub-headers to Discussion.
- Added another level of headers to Results and Methods.
- Added a figure (Fig 1) and a supplementary table (Table S8).

Response to Reviewer #1:

Garg et al. investigated genome-wide, sex-specific deviations from HWE using the 1KG dataset. The study focused on common bi-allelic SNVs and showed that HWE deviations are frequently associated with problematic sequence contexts. The authors further highlighted several reliable SNVs that exhibit heterogeneity across sexes or populations. Overall, this is a solid data analysis that could provide valuable insights for future GWAS and population genetic studies. However, the manuscript currently suffers from a lack of thematic focus. It remains unclear whether the work is intended as a methods paper, a resource/QC resource, or a conceptual piece on the interpretation of HWE in the era of modern WGS. At present, the manuscript attempts to address all three areas without a sufficiently defined central thesis.

Author response: We thank the reviewer for their comments.

Major Comments:

1. The Introduction needs to be more concise and focused. While it touches upon many relevant concepts—T2T alignment, sex-aware references, HWE, genotyping errors, sdMAF, and recent admixture—the central gap in existing literature remains ill-defined. The authors should more explicitly state what was missing in previous work, why existing HWE practices are inadequate in the context of T2T/WGS data, and what specific problem this study aims to solve. Currently, the extensive background information obscures the primary scientific question.

Author response: We have extensively restructured and rewritten the Introduction to address this concern. We hope this revised Introduction provides a clearer summary of the motivations for this work and the analysis's primary focus. We have also added a new figure (Fig 1) to illustrate the main analyses.

2. The proposed framework is potentially useful, Particularly in tracking the magnitude and direction of HWD and testing for heterogeneity. However, its positioning relative to existing methods is underdeveloped. The authors should address a key practical question: In which specific scenarios does this approach outperform existing tools? While the Discussion briefly mentions methods like RUTH, a more rigorous comparison is needed to help the reader understand the unique value this framework adds to the current genomic toolbox.

Author response: We thank the reviewer for raising the connection with RUTH. We have clarified the distinction in the revised manuscript.

It is well known that ignoring ancestry or population strata can inflate false-positive rates in HWE testing. Troendle and Yu (1994) addressed this problem by proposing an omnibus stratified test that aggregates squared stratum-specific HWE statistics, Z_k^2, rather than combining signed Z_k directly. We use this Troendle and Yu approach

because, as noted in the manuscript, “a priori, one group may show excess heterozygosity (δ_k<0), while another may show heterozygote depletion (δ_k>0)”. Aggregating Z_k^2 retains evidence for HWD in this setting and avoids cancellation across groups.

RUTH is a related but distinct contribution. RUTH was designed primarily to provide a robust, scalable HWE test for diverse sequencing cohorts by accounting for population structure using individual-specific allele frequencies and, when available, for genotype uncertainty using genotype likelihoods.

In its method comparison, RUTH included a stratified meta-analysis comparator, but this comparator used a sample-size-weighted Stouffer Z-score approach that combines signed Z_k directly. Troendle and Yu’s omnibus squared Z_k^2 approach was neither cited nor evaluated in RUTH. Thus, the meta-analysis comparator in RUTH is not the same as the Troendle and Yu stratified HWE test used in our work.

Unlike RUTH, which models ancestry-adjusted HWE deviation through a global deviation parameter, our approach estimates signed group-specific HWD measures, δ_k, and uses them both for second-order omnibus and heterogeneity testing across groups. This explicit use of δ_k provides an interpretable group-specific effect size, allowing us to identify whether HWD is shared across groups, differs in direction across groups, or is specific to particular populations or sex.

We have added the following text to the revised Discussion, lines 449-464:

“It is well known that ignoring ancestry or population strata can inflate false-positive rates in HWE testing. RUTH addresses this problem by modelling ancestry-adjusted HWE deviation using individual-specific allele frequencies and a global deviation parameter, while also incorporating genotype uncertainty through genotype likelihoods. In contrast, our approach follows the omnibus stratified framework of Troendle and Yu (1994), aggregating squared stratum-specific HWE statistics, Z_k^2, rather than the signed Z_k. This avoids cancellation when different groups deviate from HWE in opposite directions, for example, when one group shows excess heterozygosity (δ_k<0) and another shows heterozygote depletion (δ_k>0). Although RUTH included a stratified meta-analysis comparator, that comparator used a sample-size-weighted Stouffer Z-score approach and did not evaluate the Troendle and Yu omnibus approach. Thus, unlike RUTH, our approach estimates signed group-specific HWD measures, δ_k’s, and uses them both for second-order omnibus and heterogeneity testing across groups.

This provides interpretable group-specific effect sizes, allowing us to distinguish shared HWD from population- or sex-specific HWD."

3. In Lines 159–160: The claim that "there was more consistency between sexes of the same super-population than between super-populations of the same sex" appears to be an observational impression from Figure 2 rather than a quantified result. I could not find a formal statistical analysis supporting this statement. To maintain this claim, the authors should provide rigorous quantification, such as pairwise similarity/correlation analyses, clustering, or a direct statistical comparison of within-super-population vs. within-sex similarity.

Author response: We thank the reviewer for bringing this to our attention. The sentence was misplaced. We have moved it to where we discuss heterogeneity in HWD in reliable regions. We have also added a brief summary of related results from Table S7 to support the claim, lines 248-253:

"Of the 140 SNPs, 79 and 86 exhibited significant heterogeneity in HWD across the 5 super-population groups in females and males, respectively (including 58 in common). However, none of the 140 SNPs were significant across sexes in super-population groups. Additionally, 34 of the 140 SNPs were only significant in the heterogeneity test of HWD when all 10 groups were analysed together."

4. Figure 3 is significantly underexplained. The Results section mentions that the figure shows X-chromosome results but provides little further interpretation. Given that this is a primary figure, the authors must provide a more detailed walkthrough and discussion of the findings presented there.

Author response: We have improved upon our presentation of results for chromosome X, by adding lines 183-184:

"There is high HWD across super-populations: (i) at the centromeric boundaries of both PAR1 and PAR2 in males, and (ii) in the NPR region close to PAR1 in females."

See also minor comment #3 by reviewer #2, addressed below, regarding meta-analysis results in PARs.

5. The practical motivation for using T2T-CHM13v2.0 in this context is not yet fully convincing. Most GWAS are still array-based or utilize older references (e.g., hg19), and many sequencing pipelines already exclude complex or poorly mappable regions. Since a major finding of this study is that HWE deviations are concentrated in these complex regions, the authors need to be more explicit about what this work adds to current practice.

Author response: We hope our updated introduction now provides stronger support for using the T2T-CHM13v2.0-aligned 1KGP. While we agree that many GWASes today are

not yet using the newer builds, future ones likely will, including the alignment of WGS to pangenomes. We have added line 470-471 to the Discussion:

“Even though current GWASes may not be based on newer reference builds, future arrays, imputations or sequencing will likely be.”

See also comment #5 of reviewer #2 addressed below.

We have added some of the following responses in the new Recommendations sub-section of the Discussion section (lines 576-581):

“Our analysis reveals that males have high HWD at PAR boundaries, indicating that applying sex-specific HWE-based quality control in males in PARs may inadvertently remove SNPs. We found SNPs with marked deviations, despite filtering out regions that are not ‘reliable’ using several external genomic annotations. Therefore, this filtering may not be sufficient. The annotations may also be biased by the ancestral composition of genomes sequenced to date.”

Specifically:

(1) If researchers are already filtering out these regions, what should they do differently after reading this paper?

Author response: Despite filtering, we observed regions with marked heterogeneity; therefore, the filtering is insufficient. As a result, researchers should still check for HWD after filtering out these regions. As others have documented, the vast majority of genomes, historically, have been of European ancestry, and since some of the annotations are based on structural variants, similar variants that are more common in non-European populations may not yet be included in the annotations.

(2) Is the primary value in a more systematic definition of "reliable regions" for T2T-aligned WGS?

Author response: No, “reliable regions” is a context-dependent term. For example, it will be different for long-read sequencing data. However, the multivariate model may be helpful in predicting HWD for short-read WGS aligned to other builds.

(3) Does it demonstrate that sex-aware alignment still leaves significant structured artifacts?

Author response: We are not clear what is meant by ‘structured artifacts’ here. Does this mean structural variants, or does it refer to sex chromosomes or something else? Sex-aware alignment primarily benefits PARs (Webster et al. 2019); it may not prevent autosomal genotyping errors due to PSVs.

(4) Or does it identify residual outliers that survive standard filters? The authors should clearly state the "lessons learned" for practitioners to make the study's impact more apparent.

Author response: If we applied standard HWE filters to PARs, variants at the NPR boundary may be flagged as having high HWE due to marked deviations in males (see minor comment #3 by reviewer #2 addressed below).

Response to Reviewer #2:

Summary

This manuscript examines Hardy-Weinberg disequilibrium (HWD) in whole-genome sequencing data using a sex- and super-population–stratified framework applied to T2T-aligned 1000 Genomes data. The study provides a comprehensive characterization of HWD patterns across autosomes and the X chromosome and highlights the strong influence of genomic context on HWD signals. The manuscript is generally well written, and the analysis linking HWD to sequence-related genomic features is a strength. However, several aspects of the interpretation and technical analysis could be further clarified.

Author response: We thank the reviewer for their comments.

Major Comments

1. Interpretation of the omnibus and heterogeneity analyses
The manuscript presents an omnibus meta-analysis and a heterogeneity test across the 10 sex-by-super-population groups, but the relationship between these two analyses is not clearly explained. In particular, many SNPs that are significant in the omnibus analysis also show significant heterogeneity, both before and after restriction to reliable regions. This makes it difficult to interpret what omnibus significance represents, for example whether it reflects broadly consistent deviations across groups or is driven by a subset of groups with stronger effects.

Author response: We have now clarified the significance of heterogeneity in results, lines 164-165 :

"About half of the significant SNPs in the omnibus meta-analysis also showed significant HWE heterogeneity, which signifies unequal contribution of groups."

Table 2 provides the total number of SNPs in both cases. We have done more detailed analyses for the reliable regions. Table S7 shows SNP-level detail, including additional heterogeneity by super-population/sex. Fig 7 provides a clear graphical representation of the patterns observed across sexes and super-populations. We have added a new Fig 1 to provide an overview of the analysis structure with a focus on HWE heterogeneity.

Additional details on the meta-analysis illustrate that the omnibus signal at PAR-NPR boundaries is heterogenous, driven predominantly by males; see minor comment #3 addressed below and comment #3 by reviewer #1 addressed above.

2. Interpretation of signals in reliable regions

Restricting the analysis to reliable regions substantially reduces the number of significant SNPs, which is an important result. However, a subset of signals remains

after filtering, and their interpretation is not fully clear. These loci often show heterozygote excess and, in some cases, substantial heterogeneity across groups, raising questions about whether they reflect residual technical or sequence-related effects or other factors not captured by the current definition of reliable regions.

The chr14 locus discussed in the manuscript illustrates this point. While several possible explanations are considered, the broader implications for interpreting such loci and for understanding the limitations of the current filtering strategy are not fully developed. Expanding this discussion would strengthen the manuscript.

Author response: Loci with omnibus significance in reliable regions need to be analysed individually to assess what is driving the HWD. We have done an in-depth analysis of the chr14 locus, including checking allele depth (Fig S8-10). We have also now added a brief analysis of chr19 and chr20 loci.

However, without long-read sequencing and/or pangenomes, we cannot be sure if the HWD in reliable regions is an artifact of short-read sequencing. We have now added lines 570-571 to the new Limitations sub-section of the Discussion.

"Short-read whole genome sequencing from cell-lines can have more artifacts (Byrska-Bishop et al. 2022), which may be resolved by using long-read sequencing of non-transformed cells."

See also comment #3 by reviewer #1 addressed above.

3. Evaluation of the multivariate feature model

The multivariate feature model is an important component of the study. While the reported AUC is high, the evaluation is based on training and testing within the same dataset, using odd versus even autosomes after interval-based thinning. As a result, the training and test sets remain closely matched in their underlying data characteristics, making it difficult to assess how well the model generalizes beyond this dataset.

Author response: We thank the reviewer for noting this. We have now added lines 571-574 to the new Limitations sub-section of the Discussion.

"Although we trained and tested the multivariate model on different chromosomes, the underlying data was the same. Currently, due to a lack of other large short-read sequencing data aligned to v2, we have not tested it on an external dataset."

4. Interpretation of admixture-related HWD

The section on recently admixed populations is interesting, but its interpretation could be clarified. The results show heterozygote excess in autosomes, whereas heterozygote deficiency is often observed under population substructure (e.g., the

Wahlund effect). It is not clear how this difference in direction should be interpreted. Clarifying this point would strengthen the interpretation.

Author response: We thank the reviewer for bringing this to our attention. We have now addressed it in the Discussion, lines 547-550:

"This is in contrast to what we expect when two sub-populations have non-random mating, which results in paucity of heterozygosity (i.e. Wahlund effect). Relatively recent admixture and/or non-random mating can result in heterozygote excess on autosomes."

We have conducted further analysis of the autosomal SNPs that deviate in recently admixed populations and found that they all overlap with the SNPs identified in the primary omnibus meta-analysis, which also exhibit heterozygote excess. Therefore, this observation is not unique to the recently-admixed analysis. We have discussed the phenomenon of heterozygote excess in other parts of the Discussion, for example, with regard to the chr14 locus. The figure below shows a genome-wide Manhattan plot of HWE in recently-admixed populations (lines 315-316).

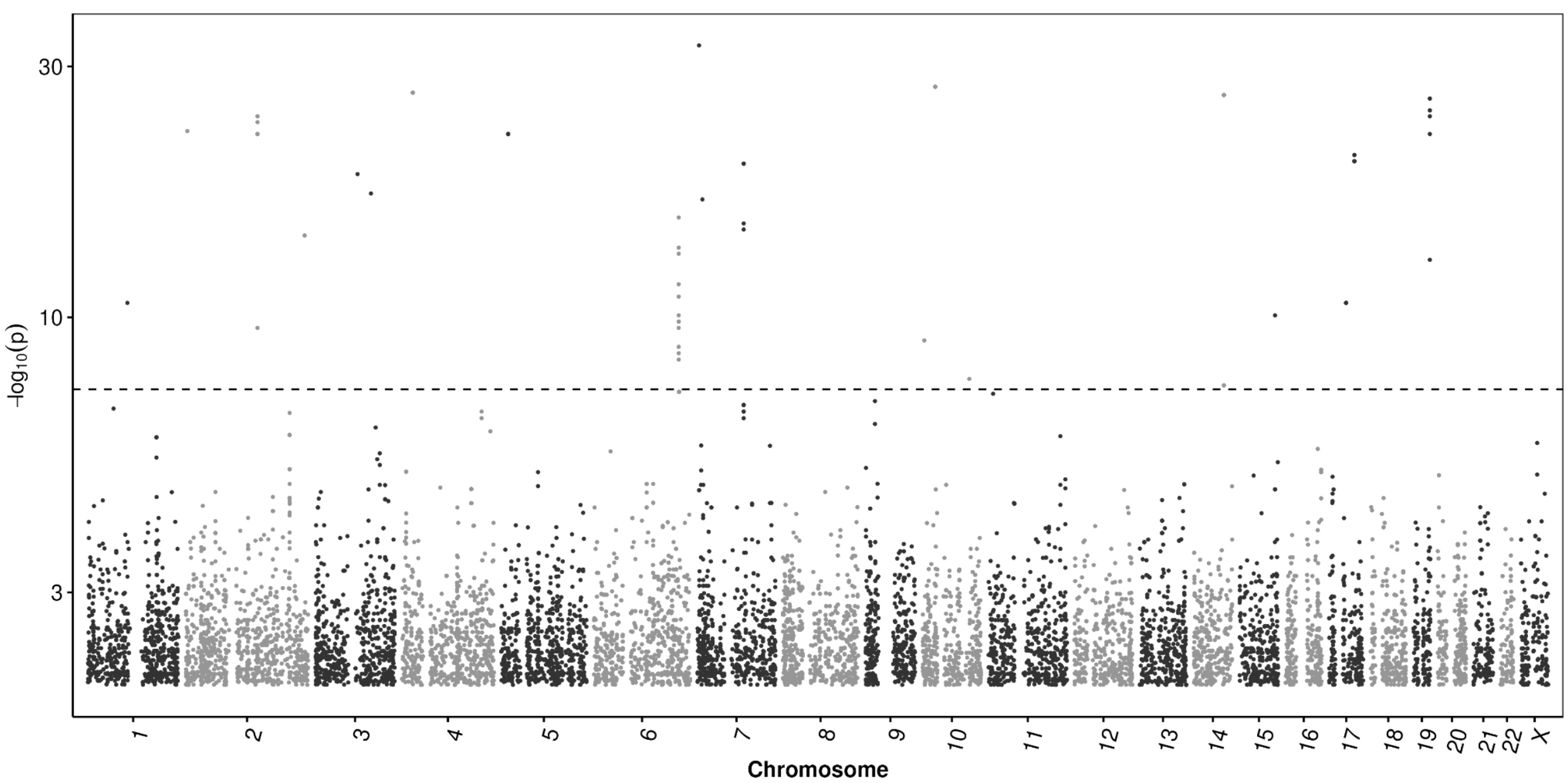

5. Role of the T2T reference

The study utilizes a T2T-based dataset, but it is not yet clear what the use of the T2T reference adds to the analysis of HWE/HWD. While the manuscript shows extensive HWD in challenging regions and a strong reduction after restricting to reliable regions, it is unclear whether these patterns are specific to T2T or reflect general properties of the data.

Author response: T2T adds around 200 Mb (equivalent to adding another chromosome 3) of sequence to GRCh38, which has otherwise not been subjected to

HWE. Aganezov et al. 2022 performed a detailed comparison of T2T and hg38 specifically in the context of 1KGP data, but they did not examine HWE in the same manner that we do.

One of the regions we have looked at in depth is a chr14 locus (Fig 7, S7-10), which we have also examined in the hg38 version (the same 1KGP data aligned to GRCh38). We observed a similar pattern of allele depth across T2T and hg38 versions (Figure S9), indicating that this locus is a general property of the data rather than specific to T2T. To perform a systematic comparison between builds would require a parallel analysis of genome-wide HWE in hg38.

T2T data can also be compared with pangenomes in the future to determine whether the patterns are build-specific. We have added lines 537-538 to the Discussion:

“Future analysis of additional pangenome samples may provide insight into deviation in such regions.”

See also comment #5 by reviewer #1 addressed above.

6. Clarity of key conclusions

The manuscript contains substantial analysis, but in several sections the key conclusions are not clearly emphasized. For example, in the discussion of SNPs in reliable regions and in the omnibus and heterogeneity analyses, multiple observations and specific examples are presented in sequence, which can make the main points less clear. More clearly highlighting the key conclusions would help bring out the main findings.

Author response: We have revised the manuscript to address this concern, including extensive restructuring of the Introduction and Discussion sections. We have added another level of headings in the Results section to make it easier to follow. We hope this version better highlights the key conclusions.

Minor comments

1. The manuscript introduces the concept of “reliable regions,” which plays a central role in downstream analyses. A clearer summary of this definition in the main text would improve readability.

Author response: We provided the full list of genomic features that constitute the “reliable regions” in Methods (Table S12). We have now introduced the phrase earlier in lines 102-104:

“We have combined several of these genomic features to create a set of ‘reliable regions’, which we use as a filter prior to our main analyses.”

2. When discussing the 255 SNPs identified in reliable regions, the manuscript presents detailed descriptive results, including their distribution, heterogeneity, and group-specific patterns. However, the main points are not immediately clear. Clarifying this section would make the key findings easier to follow.

Author response: See comment #3 by reviewer #1 addressed above.

3. The elevated HWD observed near PAR–NPR boundaries, particularly in males, is notable. Given the known complexity of these regions, including X/Y homology and ploidy-related calling challenges, it would be helpful to discuss the potential influence of technical factors.

Author response: As described in the Introduction, the v2 data comes from sex-chromosome-aware alignment, in which samples are aligned to only X-PARs by masking Y-PARs (Webster et al. 2019).

Since the read length for paired-end Illumina sequencing is approximately 150bp, reads that span the PAR-NPR boundary would only be expected to interfere with variant calling in males over a short distance. In this dataset, HWD in males on PAR1 and PAR2 occurs as close as 160 bp and as far as 4,138 bp from the boundary, respectively. Given the sex-chromosome-aware alignment of this data, and the fact that the majority of the deviating variants are far from the boundary, we do not think this is a variant calling issue.

We have provided further details on SNPs with omnibus HWD in PARs in a new table (S8) and added lines 277-288:

“Table S8 provides meta-analysis results for 44 SNPs in PAR1 and 31 SNPs in PAR2, which have significant omnibus HWD after removing difficult-to-sequence regions. In contrast to the autosomes, the PARs exhibited greater similarity in HWD patterns across super-populations than between sexes, essentially because males showed higher HWD near the PAR–NPR boundaries. At the boundary of PAR1 and NPR (2-2.5Mb on chromosome X), 9 SNPs were omnibus HWD significant (Fig 5C), of which 7 also demonstrated significant heterogeneity in HWD. Further analysis indicated that all of them were heterogenous across super-populations within males. Moreover, 5 of them were heterogenous between sexes in AFR, 2 in EAS and 1 in EUR, with all showing significant deviation only in males (Fig 4). Similarly, in PAR2, 31 SNPs were omnibus HWD significant (Fig 5C), and all of them also showed significant heterogeneity, with HWD present in males.”

Response Reviewer #3:

In this study, the authors conducted the HWE test in T2T 1KG data in various ways, such as sex-stratified and population-specific (five major groups) splits. Both single data set and meta-analysis were conducted in the 1KG data set. The results were largely indicative that the quality of sequencing (poor sequence quality) led to the majority of the HW deviation.

This study should be considered their continuous efforts in HWE of the authors, ranging from development of methods and to other various applications in different datasets (Ref 11, 17, 21, etc…). This paper provides a much deeper understanding of the quality of 1KG in terms of HWE tests.

As the topic is traditional and its interpretation is straight, there is no substantial obstacle in understanding the materials and the results. Only some points are given that may help improve the presentation of the manuscript

Author response: We thank the reviewer for their comments.

1 As HWD itself is as an indicator for biological signals—such as selection, masking unreliable regions surely turned off many poor-quality driven HWD but may overkill. This paper is emphasised on the poor quality issue—a device for QC. However, when comparing Fig2C and Fig2D, it seems something interesting near chrs18~19 in both meta and heterogeneity tests, could the authors look closely if they are true signals possibly. In particular, whether the signal is overall driven by all cohorts together or by a single cohort – heterogeneity. If it is former, it may be rare if a selection signal exists across cohorts, otherwise a possible selection if it is latter.

Author response: We agree that SNPs with HWD should be examined further rather than removed based on a global threshold. Combining HWE omnibus meta-analysis and heterogeneity tests could be a solution.

Assuming that the reviewer meant chr19-20, we have expanded on their results in the text (lines 272-275). Unlike the chr14 locus, we did not find any other interesting elements at these loci to report.

"Two other regions are of note (Table S7 and Fig 7): (i) 7 SNPs at chr20:1,628,096-1,628,851 in intron 1 of SIRPB1 with high HWD in both AMR males and females, and EUR females; (ii) 7 SNPs at chr19:59,586,669-59,586,716 with excess heterozygosity in all 10 groups (HWD delta ranging from -0.12 to -0.23)."

2 It seems that the unreliable ones filtered off are largely those that have been sequenced after T2T 1KG. Fig5 is interesting to classify HDW into variant features. One of my concerns is that if HWD had been employed as a caretaker in assessing the quality score for T2T 1KG. Probably not I'm wrong, but could you double check the composition of the score of those "poor quality sequence"?

Author response: If the reviewer meant that the unreliable ones are part of the ~200 Mb present in T2Tv2 but not in GRCh38, then that is incorrect. Two-thirds of all SNPs in v2 are in 'unreliable' regions. Table S3 shows 5 SNPs in reliable regions with HWD with no coordinates for GRCh38 (column 5), indicating that these are exclusively in T2Tv2. The bioinformatic pipeline used by the authors of v2 resource does not explicitly mention HWE-based filters (https://github.com/schatzlab/t2t-chm13-chry/blob/main/alignment_variant_calling_pipeline/README.md). We have only analyzed the variants that pass their VQSR filter, as described in Methods (lines 322-327).

We are unclear about what the reviewer meant by 'score'. We have shown multiple VCF QC metrics for the chr14 locus that we investigated in depth (Fig S10).

3 Test statistic (Line 362) T_D can be biased if MAF is small and sample size is small. So, MAF may be an issue in assessing the p-value of the data. It is a known issue as Fisher's exact test should be considered for small sample or low MAF (Ref 33). We need some discussion for this point.

Author response: We thank the reviewer for raising this point. We agree that the asymptotic χ^2_1 approximation for T_D may be inaccurate when MAF is low, or the sample size is small, because the genotype-count distribution is discrete and may be highly skewed. Exact HWE tests are often preferable in such settings because they avoid relying on the asymptotic approximation and provide better finite-sample calibration. We have now clarified this limitation in the Discussion.

In the present study, our primary analyses were restricted to SNPs with MAF≥5% in all sex-by-super-population groups, which reduces but does not eliminate this concern. We retained T_D as the primary statistic because it is based on the signed HWD measure δ, which is essential for distinguishing excess heterozygosity from heterozygote depletion and for conducting our second-order omnibus and heterogeneity analyses across groups. We now note that exact tests may be useful as complementary sensitivity analyses, especially for rare variants, small strata, or variants near the MAF threshold.

We have added the following text to the revised manuscript, lines 557-568:

"The statistic TDT_{DTD} relies on an asymptotic χ^2_1 approximation, which may be inaccurate when MAF is low, or the sample size is small. Exact HWE tests avoid this asymptotic approximation and can provide better finite-sample calibration, although exact p-values are not necessarily more conservative than chi-square p-values for every observed dataset. In this study, we reduced this concern by restricting the primary analyses to SNPs with MAF≥5% in all 10 groups (super-populations x sexes), but finite-sample calibration may still be imperfect for variants near the MAF threshold. We retained T_D because it is based on the signed HWD measure δ, which allows us to distinguish excess heterozygosity from heterozygote depletion and to perform

second-order omnibus and heterogeneity analyses across groups. Exact tests may therefore be useful as complementary sensitivity analyses, particularly for rare variants or smaller strata."

4 Line 321-324: Recent admixed samples (ASW) are very difficult to tackle in HWE. As reported, the global LD of admixed samples are very different from super-populations. LD and HWE/Fst are two sides of the same coin (eLife, 2023, 12:90636), otherwise the baseline inflation, for either HWE (Fst) or LD, could be justified, the test statistics are too aggressive for sure in assessing ASW.

Author response: We checked with the authors of 'https://elifesciences.org/articles/90636' about which version of 1KGP they used. They cited 'https://wellcomeopenresearch.org/articles/4-50/v2', which used phase 3 data and consensus calls from multiple methods (with a minimum depth of ~9X outside the exome). So these observations derive from low-coverage WGS and exome data and, to our knowledge, have not been examined in high-coverage data. Also, note that we have used ASW and ACB samples together in our analysis.
See also comment #4 by reviewer #2 addressed above.

5 the font size of legends are too small and sub-titles are too small for clear illustration. Please consider remake the illustration

Author response: We have moved all figure legends to the end of the main text and changed their font size. We have also now provided separate files for the main figures. We hope the reformatting in accordance with PLOS guidelines has also made them more legible.

6 Lines 182, 197: "SNPS", typo.

Author response: Thanks for pointing these out. We have fixed the typos.

7 Lines 238~242 text about Fig2c-d bumped up sudden and stood far away from its Fig2a-b. There are subsections in Introduction but no any subsections in Results. Some editorials are needed.

Author response: We have removed figures from the main text. Assuming the reviewer meant adding sub-sections to the Discussion, because Results already have them, we thank them for the suggestion and have added sub-headers. We have also improved upon the structure of the Introduction and Discussion.

8 Of note, HWE could not be tested in one generation but a by-generation process. The HWE test of this paper is actually a test for Hardy-Weinberg proportion/frequencies given the estimated allele frequency (see Lynch&Walsh 1998, Genetics and Analysis of Quantitative Traits, page 55). In recently literatures, these two procedures are merged to the same one.

Author response: We agree that multi-generational data can provide additional information about HWE, but it is typically uncommon in most cohorts.
