# Supplementary material for: Assessing Hardy-Weinberg equilibrium in T2T-aligned 1000 genomes project
Source: PLoS Genet. 2026 Sep 15;22(9):e1012280. doi: 10.1371/journal.pgen.1012280 (PMC13596830; doi:10.1371/journal.pgen.1012280)
Supplement: Peer review history

# Peer review: Decision Letter 0

**DOI:** 10.1371/journal.pgen.1012280.r001

**Authors:** Hua Tang, Chaolong Wang

22 Apr 2026

PGENETICS-D-26-00164

Assessing Hardy-Weinberg Equilibrium in T2T-aligned 1000 Genomes Project

PLOS Genetics

Dear Dr. Paterson,

Thank you for submitting your manuscript to PLOS Genetics. After careful consideration, we feel that it has merit but does not fully meet PLOS Genetics's publication criteria as it currently stands. Therefore, we invite you to submit a revised version of the manuscript that addresses the points raised during the review process.

Please submit your revised manuscript by Jun 21 2026 11:59PM. If you will need more time than this to complete your revisions, please reply to this message or contact the journal office at plosgenetics@plos.org. When you're ready to submit your revision, log on to https://www.editorialmanager.com/pgenetics/ and select the 'Submissions Needing Revision' folder to locate your manuscript file.

Please include the following items when submitting your revised manuscript:

* A letter that responds to each point raised by the editor and reviewer(s). You should upload this letter as a separate file labeled 'Response to Reviewers'. This file does not need to include responses to any formatting updates and technical items listed in the 'Journal Requirements' section below.

* A marked-up copy of your manuscript that highlights changes made to the original version. You should upload this as a separate file labeled 'Revised Manuscript with Track Changes'.

* An unmarked version of your revised paper without tracked changes. You should upload this as a separate file labeled 'Manuscript'.

If you would like to make changes to your financial disclosure, competing interests statement, or data availability statement, please make these updates within the submission form at the time of resubmission. Guidelines for resubmitting your figure files are available below the reviewer comments at the end of this letter.

We look forward to receiving your revised manuscript.

Kind regards,

Chaolong Wang

Academic Editor

PLOS Genetics

Hua Tang

Section Editor

PLOS Genetics

Aimée Dudley

Editor-in-Chief

PLOS Genetics

Anne Goriely

Editor-in-Chief

PLOS Genetics

**Journal Requirements:**

If the reviewer comments include a recommendation to cite specific previously published works, please review and evaluate these publications to determine whether they are relevant and should be cited. There is no requirement to cite these works unless the editor has indicated otherwise.

1) Please ensure that the CRediT author contributions listed for every co-author are completed accurately and in full.

At this stage, the following Authors/Authors require contributions: Andrew D Paterson. Please ensure that the full contributions of each author are acknowledged in the "Add/Edit/Remove Authors" section of our submission form.

The list of CRediT author contributions may be found here: https://journals.plos.org/plosgenetics/s/authorship#loc-author-contributions

2) We ask that a manuscript source file is provided at Revision. Please upload your manuscript file as a .doc, .docx, .rtf or .tex. If you are providing a .tex file, please upload it under the item type u2018LaTeX Source Fileu2019 and leave your .pdf version as the item type u2018Manuscriptu2019.

3) Please upload all main figures as separate Figure files in .tif or .eps format. For more information about how to convert and format your figure files please see our guidelines:

https://journals.plos.org/plosgenetics/s/figures

4) We have noticed that you have uploaded Supporting Information files, but you have not included a list of legends. Please add a full list of legends for your Supporting Information files after the references list.

5) Please amend your detailed Financial Disclosure statement. This is published with the article. It must therefore be completed in full sentences and contain the exact wording you wish to be published.

1) State the initials, alongside each funding source, of each author to receive each grant. For example: "This work was supported by the National Institutes of Health (####### to AM; ###### to CJ) and the National Science Foundation (###### to AM)."

2) State what role the funders took in the study. If the funders had no role in your study, please state: "The funders had no role in study design, data collection and analysis, decision to publish, or preparation of the manuscript."

3) If any authors received a salary from any of your funders, please state which authors and which funders..

If you did not receive any funding for this study, please simply state: u201cThe authors received no specific funding for this work.u201d

**Reviewers' comments:**

Reviewer's Responses to Questions

**Comments to the Authors:**

**Please note here if the review is uploaded as an attachment.**

Reviewer #1: Garg et al. investigated genome-wide, sex-specific deviations from HWE using the 1KG dataset. The study focused on common bi-allelic SNVs and showed that HWE deviations are frequently associated with problematic sequence contexts. The authors further highlighted several reliable SNVs that exhibit heterogeneity across sexes or populations. Overall, this is a solid data analysis that could provide valuable insights for future GWAS and population genetic studies. However, the manuscript currently suffers from a lack of thematic focus. It remains unclear whether the work is intended as a methods paper, a resource/QC resource, or a conceptual piece on the interpretation of HWE in the era of modern WGS. At present, the manuscript attempts to address all three areas without a sufficiently defined central thesis.

Major Comments:

1. The Introduction needs to be more concise and focused. While it touches upon many relevant concepts—T2T alignment, sex-aware references, HWE, genotyping errors, sdMAF, and recent admixture—the central gap in existing literature remains ill-defined. The authors should more explicitly state what was missing in previous work, why existing HWE practices are inadequate in the context of T2T/WGS data, and what specific problem this study aims to solve. Currently, the extensive background information obscures the primary scientific question.

2. The proposed framework is potentially useful, Particularly in tracking the magnitude and direction of HWD and testing for heterogeneity. However, its positioning relative to existing methods is underdeveloped. The authors should address a key practical question: In which specific scenarios does this approach outperform existing tools? While the Discussion briefly mentions methods like RUTH, a more rigorous comparison is needed to help the reader understand the unique value this framework adds to the current genomic toolbox.

3. In Lines 159–160: The claim that "there was more consistency between sexes of the same super-population than between super-populations of the same sex" appears to be an observational impression from Figure 2 rather than a quantified result. I could not find a formal statistical analysis supporting this statement. To maintain this claim, the authors should provide rigorous quantification, such as pairwise similarity/correlation analyses, clustering, or a direct statistical comparison of within-super-population vs. within-sex similarity.

4. Figure 3 is significantly underexplained. The Results section mentions that the figure shows X-chromosome results but provides little further interpretation. Given that this is a primary figure, the authors must provide a more detailed walkthrough and discussion of the findings presented there.

5. The practical motivation for using T2T-CHM13v2.0 in this context is not yet fully convincing. Most GWAS are still array-based or utilize older references (e.g., hg19), and many sequencing pipelines already exclude complex or poorly mappable regions. Since a major finding of this study is that HWE deviations are concentrated in these complex regions, the authors need to be more explicit about what this work adds to current practice. Specifically: (1)If researchers are already filtering out these regions, what should they do differently after reading this paper? (2) Is the primary value in a more systematic definition of "reliable regions" for T2T-aligned WGS? (3) Does it demonstrate that sex-aware alignment still leaves significant structured artifacts? (4) Or does it identify residual outliers that survive standard filters? The authors should clearly state the "lessons learned" for practitioners to make the study's impact more apparent.

Reviewer #2: Summary

This manuscript examines Hardy-Weinberg disequilibrium (HWD) in whole-genome sequencing data using a sex- and super-population–stratified framework applied to T2T-aligned 1000 Genomes data. The study provides a comprehensive characterization of HWD patterns across autosomes and the X chromosome and highlights the strong influence of genomic context on HWD signals. The manuscript is generally well written, and the analysis linking HWD to sequence-related genomic features is a strength. However, several aspects of the interpretation and technical analysis could be further clarified.

Major Comments

1. Interpretation of the omnibus and heterogeneity analyses

The manuscript presents an omnibus meta-analysis and a heterogeneity test across the 10 sex-by-super-population groups, but the relationship between these two analyses is not clearly explained. In particular, many SNPs that are significant in the omnibus analysis also show significant heterogeneity, both before and after restriction to reliable regions. This makes it difficult to interpret what omnibus significance represents, for example whether it reflects broadly consistent deviations across groups or is driven by a subset of groups with stronger effects.

2. Interpretation of signals in reliable regions

Restricting the analysis to reliable regions substantially reduces the number of significant SNPs, which is an important result. However, a subset of signals remains after filtering, and their interpretation is not fully clear. These loci often show heterozygote excess and, in some cases, substantial heterogeneity across groups, raising questions about whether they reflect residual technical or sequence-related effects or other factors not captured by the current definition of reliable regions.

The chr14 locus discussed in the manuscript illustrates this point. While several possible explanations are considered, the broader implications for interpreting such loci and for understanding the limitations of the current filtering strategy are not fully developed. Expanding this discussion would strengthen the manuscript.

3. Evaluation of the multivariate feature model

The multivariate feature model is an important component of the study. While the reported AUC is high, the evaluation is based on training and testing within the same dataset, using odd versus even autosomes after interval-based thinning. As a result, the training and test sets remain closely matched in their underlying data characteristics, making it difficult to assess how well the model generalizes beyond this dataset.

4. Interpretation of admixture-related HWD

The section on recently admixed populations is interesting, but its interpretation could be clarified. The results show heterozygote excess in autosomes, whereas heterozygote deficiency is often observed under population substructure (e.g., the Wahlund effect). It is not clear how this difference in direction should be interpreted. Clarifying this point would strengthen the interpretation.

5. Role of the T2T reference

The study utilizes a T2T-based dataset, but it is not yet clear what the use of the T2T reference adds to the analysis of HWE/HWD. While the manuscript shows extensive HWD in challenging regions and a strong reduction after restricting to reliable regions, it is unclear whether these patterns are specific to T2T or reflect general properties of the data.

6. Clarity of key conclusions

The manuscript contains substantial analysis, but in several sections the key conclusions are not clearly emphasized. For example, in the discussion of SNPs in reliable regions and in the omnibus and heterogeneity analyses, multiple observations and specific examples are presented in sequence, which can make the main points less clear. More clearly highlighting the key conclusions would help bring out the main findings.

Minor comments

1. The manuscript introduces the concept of “reliable regions,” which plays a central role in downstream analyses. A clearer summary of this definition in the main text would improve readability.

2. When discussing the 255 SNPs identified in reliable regions, the manuscript presents detailed descriptive results, including their distribution, heterogeneity, and group-specific patterns. However, the main points are not immediately clear. Clarifying this section would make the key findings easier to follow.

3. The elevated HWD observed near PAR–NPR boundaries, particularly in males, is notable. Given the known complexity of these regions, including X/Y homology and ploidy-related calling challenges, it would be helpful to discuss the potential influence of technical factors.

Reviewer #3: In this study, the authors conducted the HWE test in T2T 1KG data in various ways, such as sex-stratified and population-specific (five major groups) splits. Both single data set and meta-analysis were conducted in the 1KG data set. The results were largely indicative that the quality of sequencing (poor sequence quality) led to the majority of the HW deviation.

This study should be considered their continuous efforts in HWE of the authors, ranging from development of methods and to other various applications in different datasets (Ref 11, 17, 21, etc…). This paper provides a much deeper understanding of the quality of 1KG in terms of HWE tests.

As the topic is traditional and its interpretation is straight, there is no substantial obstacle in understanding the materials and the results. Only some points are given that may help improve the presentation of the manuscript

1 As HWD itself is as an indicator for biological signals—such as selection, masking unreliable regions surely turned off many poor-quality driven HWD but may overkill. This paper is emphasised on the poor quality issue—a device for QC. However, when comparing Fig2C and Fig2D, it seems something interesting near chrs18~19 in both meta and heterogeneity tests, could the authors look closely if they are true signals possibly. In particular, whether the signal is overall driven by all cohorts together or by a single cohort – heterogeneity. If it is former, it may be rare if a selection signal exists across cohorts, otherwise a possible selection if it is latter.

2 It seems that the unreliable ones filtered off are largely those that have been sequenced after T2T 1KG. Fig5 is interesting to classify HDW into variant features. One of my concerns is that if HWD had been employed as a caretaker in assessing the quality score for T2T 1KG. Probably not I’m wrong, but could you double check the composition of the score of those “poor quality sequence”?

3 Test statistic (Line 362) T_D can be biased if MAF is small and sample size is small. So, MAF may be an issue in assessing the p-value of the data. It is a known issue as Fisher’s exact test should be considered for small sample or low MAF (Ref 33). We need some discussion for this point.

4 Line 321-324: Recent admixed samples (ASW) are very difficult to tackle in HWE. As reported, the global LD of admixed samples are very different from super-populations. LD and HWE/Fst are two sides of the same coin (eLife, 2023, 12:90636), otherwise the baseline inflation, for either HWE (Fst) or LD, could be justified, the test statistics are too aggressive for sure in assessing ASW.

5 the font size of legends are too small and sub-titles are too small for clear illustration. Please consider remake the illustration

6 Lines 182, 197: “SNPS”, typo.

7 Lines 238~242 text about Fig2c-d bumped up sudden and stood far away from its Fig2a-b. There are subsections in Introduction but no any subsections in Results. Some editorials are needed.

8 Of note, HWE could not be tested in one generation but a by-generation process. The HWE test of this paper is actually a test for Hardy-Weinberg proportion/frequencies given the estimated allele frequency (see Lynch&Walsh 1998, Genetics and Analysis of Quantitative Traits, page 55). In recently literatures, these two procedures are merged to the same one.

**********

**Have all data underlying the figures and results presented in the manuscript been provided?**

Large-scale datasets should be made available via a public repository as described in the *PLOS Genetics*  data availability policy, and numerical data that underlies graphs or summary statistics should be provided in spreadsheet form as supporting information.

Reviewer #1: Yes

Reviewer #2: Yes

Reviewer #3: Yes

**********

PLOS authors have the option to publish the peer review history of their article (what does this mean?). If published, this will include your full peer review and any attached files.

If you choose “no”, your identity will remain anonymous but your review may still be made public.

**Do you want your identity to be public for this peer review?**  For information about this choice, including consent withdrawal, please see our Privacy Policy.

Reviewer #1: No

Reviewer #2: No

Reviewer #3: No

**Figure resubmission:**

While revising your submission, we strongly recommend that you use PLOS’s NAAS tool (https://ngplosjournals.pagemajik.ai/artanalysis) to test your figure files. NAAS can convert your figure files to the TIFF file type and meet basic requirements (such as print size, resolution), or provide you with a report on issues that do not meet our requirements and that NAAS cannot fix.

After uploading your figures to PLOS’s NAAS tool - https://ngplosjournals.pagemajik.ai/artanalysis, NAAS will process the files provided and display the results in the "Uploaded Files" section of the page as the processing is complete. If the uploaded figures meet our requirements (or NAAS is able to fix the files to meet our requirements), the figure will be marked as "fixed" above. If NAAS is unable to fix the files, a red "failed" label will appear above. When NAAS has confirmed that the figure files meet our requirements, please download the file via the download option, and include these NAAS processed figure files when submitting your revised manuscript.

**Reproducibility:**

To enhance the reproducibility of your results, we recommend that authors of applicable studies deposit laboratory protocols in protocols.io, where a protocol can be assigned its own identifier (DOI) such that it can be cited independently in the future. Additionally, PLOS ONE offers an option to publish peer-reviewed clinical study protocols. Read more information on sharing protocols at https://plos.org/protocols?utm_medium=editorial-email&utm_source=authorletters&utm_campaign=protocols

# Author comment: Author response to Decision Letter 1

**DOI:** 10.1371/journal.pgen.1012280.r002

22 Jun 2026

Attachment Submitted filename: Response to reviewers.pdf

# Peer review: Decision Letter 1

**DOI:** 10.1371/journal.pgen.1012280.r003

**Authors:** Hua Tang, Chaolong Wang

10 Aug 2026

Dear Dr Paterson,

We are pleased to inform you that your manuscript entitled "Assessing Hardy-Weinberg Equilibrium in T2T-aligned 1000 Genomes Project" has been editorially accepted for publication in PLOS Genetics. Congratulations!

Before your submission can be formally accepted and sent to production you will need to complete our formatting changes, which you will receive in a follow up email. Please be aware that it may take several days for you to receive this email; during this time no action is required by you. Please note: the accept date on your published article will reflect the date of this provisional acceptance, but your manuscript will not be scheduled for publication until the required changes have been made.

Once your paper is formally accepted, an uncorrected proof of your manuscript will be published online ahead of the final version, unless you’ve already opted out via the online submission form. If, for any reason, you do not want an earlier version of your manuscript published online or are unsure if you have already indicated as such, please let the journal staff know immediately at plosgenetics@plos.org.

In the meantime, please log into Editorial Manager at https://www.editorialmanager.com/pgenetics/, click the "Update My Information" link at the top of the page, and update your user information to ensure an efficient production and billing process. Note that PLOS requires an ORCID iD for all corresponding authors. Therefore, please ensure that you have an ORCID iD and that it is validated in Editorial Manager. To do this, go to ‘Update my Information’ (in the upper left-hand corner of the main menu), and click on the Fetch/Validate link next to the ORCID field.  This will take you to the ORCID site and allow you to create a new iD or authenticate a pre-existing iD in Editorial Manager.

If you have a press-related query, or would like to know about making your underlying data available (as you will be aware, this is required for publication), please see the end of this email. If your institution or institutions have a press office, please notify them about your upcoming article at this point, to enable them to help maximise its impact. Inform journal staff as soon as possible if you are preparing a press release for your article and need a publication date.

Thank you again for supporting open-access publishing; we are looking forward to publishing your work in PLOS Genetics!

Yours sincerely,

Chaolong Wang

Academic Editor

PLOS Genetics

Hua Tang

Section Editor

PLOS Genetics

Aimée Dudley

Editor-in-Chief

PLOS Genetics

Anne Goriely

Editor-in-Chief

PLOS Genetics

www.plosgenetics.org

BlueSky: @plos.bsky.social

----------------------------------------------------

Comments from the reviewers (if applicable):

Please make minor revisions according to Reviewer #2's final comments.

Reviewer's Responses to Questions

**Comments to the Authors:**

**Please note here if the review is uploaded as an attachment.**

Reviewer #1: The revised manuscript is more focused, better structured, and clearer in its methodological positioning and practical recommendations. The expanded discussion of RUTH, the improved interpretation of X-chromosomal/PAR HWD patterns, and the added limitations regarding the multivariate model have strengthened the manuscript. I have no further concerns.

Reviewer #2: The authors have made substantial revisions and added several new analyses and clarifications. Most of my previous concerns have been addressed. I have three remaining points related to original Comments 1, 3, and 5.

Follow-up on comment 1. Interpretation of the omnibus and heterogeneity analyses

The revised manuscript clarifies the overlap between the omnibus and heterogeneity results and provides further subgroup-level detail. However, the phrase “unequal contribution of groups” remains somewhat vague. Please explain more explicitly what significance in both the omnibus and heterogeneity tests implies for the corresponding group-specific HWD results and how such loci should be interpreted.

Follow-up on comment 3. Evaluation of the multivariate feature model

The added limitation in the Discussion is helpful. However, the Results still describe the model as having “high accuracy” based on an AUC of 99.4%. Because the model was trained and tested on different chromosomes from the same dataset, this wording would benefit from some qualification to align with the limitation acknowledged in the Discussion.

Follow-up on comment 5. Role of the T2T reference

Thank you for noting that the T2T reference adds ～200 Mb of sequence and for comparing the chr14 locus across the T2T- and GRCh38-aligned datasets. However, it would be helpful to clarify the specific contribution of T2T to the present study. Although T2T includes additional and newly resolved sequences, the manuscript does not indicate whether, or to what extent, these regions are represented among the variants analyzed or the reported HWD signals. Given the prominence of T2T in the title and study design, the relevance of using the T2T reference to the analyses and conclusions should be made clearer.

Reviewer #3: thanks for addressing my concerns.

it is much improved.

**********

**Have all data underlying the figures and results presented in the manuscript been provided?**

Large-scale datasets should be made available via a public repository as described in the *PLOS Genetics*  data availability policy, and numerical data that underlies graphs or summary statistics should be provided in spreadsheet form as supporting information.

Reviewer #1: Yes

Reviewer #2: Yes

Reviewer #3: Yes

**********

PLOS authors have the option to publish the peer review history of their article (what does this mean?). If published, this will include your full peer review and any attached files.

If you choose “no”, your identity will remain anonymous but your review may still be made public.

**Do you want your identity to be public for this peer review?**  For information about this choice, including consent withdrawal, please see our Privacy Policy.

Reviewer #1: No

Reviewer #2: No

Reviewer #3: No

----------------------------------------------------

**Data Deposition**

If you have submitted a Research Article or Front Matter that has associated data that are not suitable for deposition in a subject-specific public repository (such as GenBank or ArrayExpress), one way to make that data available is to deposit it in the Dryad Digital Repository. As you may recall, we ask all authors to agree to make data available; this is one way to achieve that. A full list of recommended repositories can be found on our website.

The following link will take you to the Dryad record for your article, so you won't have to re‐enter its bibliographic information, and can upload your files directly:

http://datadryad.org/submit?journalID=pgenetics&manu=PGENETICS-D-26-00164R1

More information about depositing data in Dryad is available at http://www.datadryad.org/depositing. If you experience any difficulties in submitting your data, please contact help@datadryad.org for support.

Additionally, please be aware that our data availability policy requires that all numerical data underlying display items are included with the submission, and you will need to provide this before we can formally accept your manuscript, if not already present.

----------------------------------------------------

**Press Queries**

If you or your institution will be preparing press materials for this manuscript, or if you need to know your paper's publication date for media purposes, please inform the journal staff as soon as possible so that your submission can be scheduled accordingly. Your manuscript will remain under a strict press embargo until the publication date and time. This means an early version of your manuscript will not be published ahead of your final version. PLOS Genetics may also choose to issue a press release for your article. If there's anything the journal should know or you'd like more information, please get in touch via plosgenetics@plos.org.

# Editor report: Acceptance letter

**DOI:** 10.1371/journal.pgen.1012280.r004

**Authors:** Hua Tang, Chaolong Wang

PGENETICS-D-26-00164R1

Assessing Hardy-Weinberg Equilibrium in T2T-aligned 1000 Genomes Project

Dear Dr Paterson,

We are pleased to inform you that your manuscript entitled "Assessing Hardy-Weinberg Equilibrium in T2T-aligned 1000 Genomes Project" has been formally accepted for publication in PLOS Genetics! Your manuscript is now with our production department and you will be notified of the publication date in due course.

The corresponding author will soon be receiving a typeset proof for review, to ensure errors have not been introduced during production. Please review the PDF proof of your manuscript carefully, as this is the last chance to correct any errors. Please note that major changes, or those which affect the scientific understanding of the work, will likely cause delays to the publication date of your manuscript.

Soon after your final files are uploaded, unless you have opted out or your manuscript is a front-matter piece, the early version of your manuscript will be published online. The date of the early version will be your article's publication date. The final article will be published to the same URL, and all versions of the paper will be accessible to readers.

For Research Articles, you will receive an invoice from PLOS for your publication fee after your manuscript has reached the completed accept phase. If you receive an email requesting payment before acceptance or for any other service, this may be a phishing scheme. Learn how to identify phishing emails and protect your accounts at https://explore.plos.org/phishing.

Thank you again for supporting PLOS Genetics and open-access publishing. We are looking forward to publishing your work!

With kind regards,

Sharmila Kamatchi

PLOS Genetics

On behalf of:

The PLOS Genetics Team

Carlyle House, Carlyle Road, Cambridge CB4 3DN | United Kingdom

plosgenetics@plos.org | +44 (0) 1223-442823

plosgenetics.org | Twitter: @PLOSGenetics
